# Supplementary material for: Investigation of NPB Analogs That Target Phosphorylation of BAD-Ser99 in Human Mammary Carcinoma Cells
Source: Int J Mol Sci. 2021 Oct 12;22(20):11002. doi: 10.3390/ijms222011002 (PMC8540132; doi:10.3390/ijms222011002)
Supplement: Supplementary file 1 [file ijms-22-11002-s001.zip › ijms-1315289-supplementary.pdf]

**Investigation of NPB analogs that target phosphorylation of BAD-Ser99 in human mammary carcinoma cells**

Swamy Savvemala Girimanchanaika, Dukanya Dukanya, Ananda Swamynayak, Divya Maldepalli Govindachar, Mahendra Madegowda, Ganga Periyasamy, Kanchugarakoppal Subbegowda Rangappa, Vijay Pandey, Peter E. Lobie, Basappa Basappa

# Supplementary Information

## $^{1}H$ NMR, $^{13}C$ NMR, Mass of NPB analogs

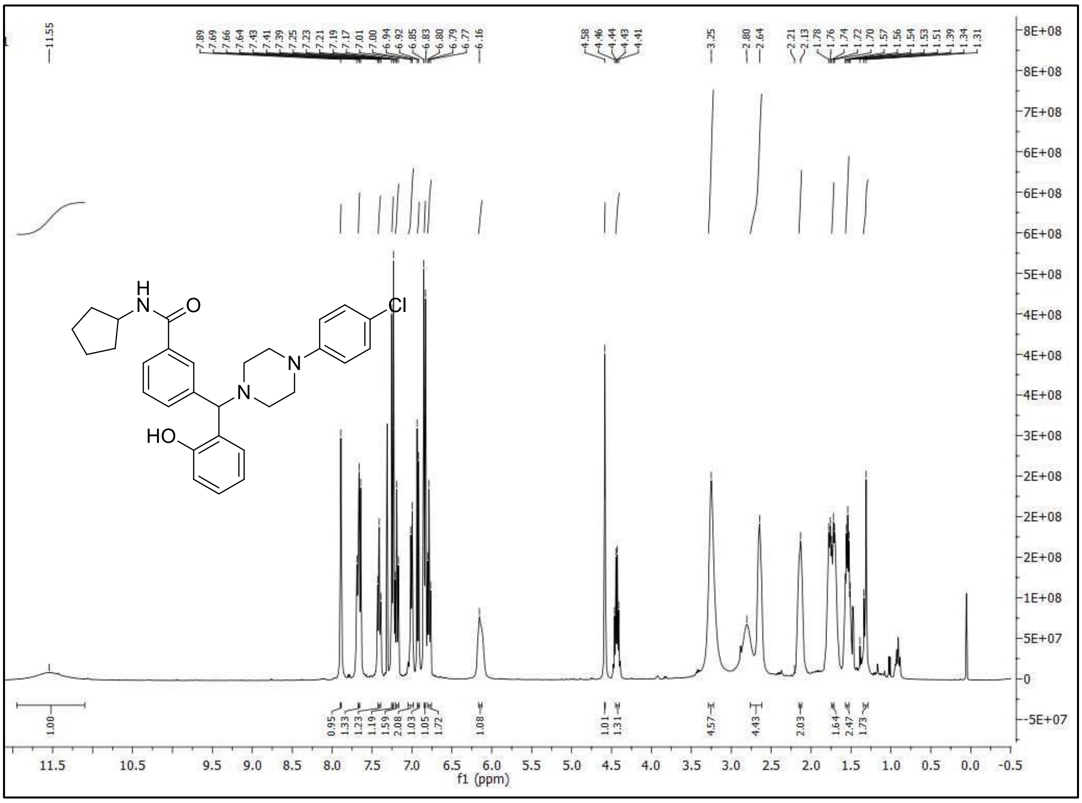

S1: $^{1}$H NMR spectrum of compound **4a**

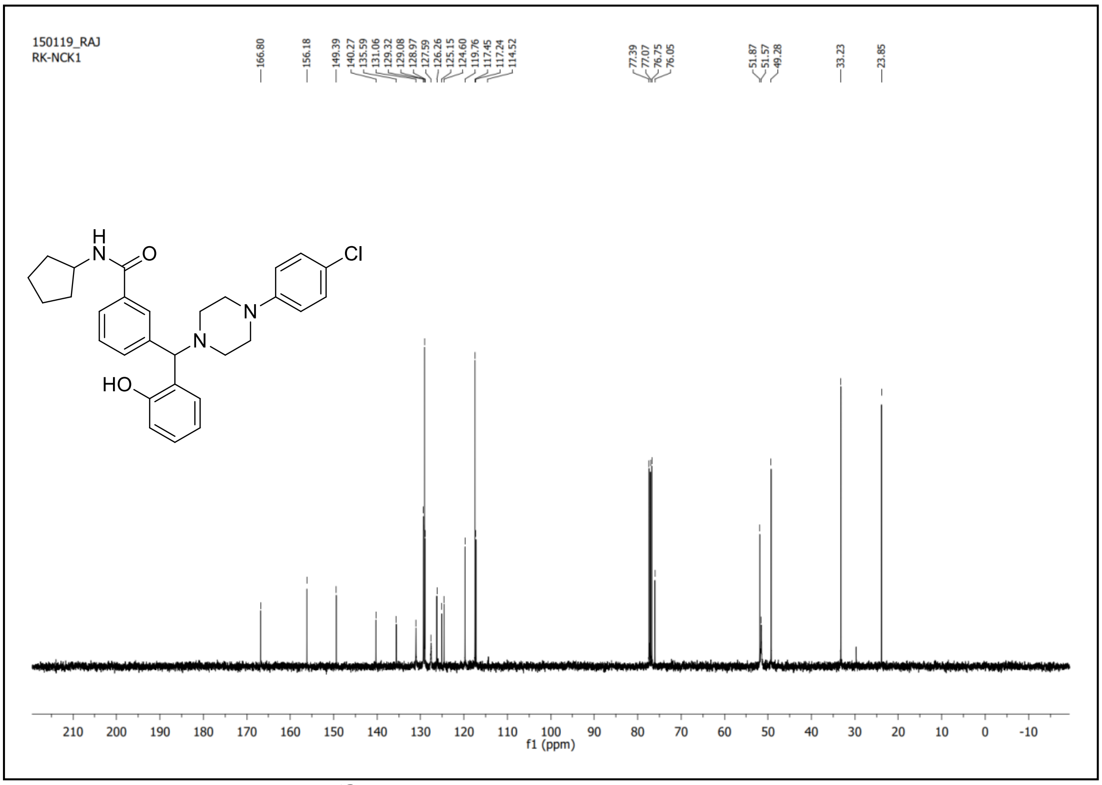


S2:$^{13}$ C NMR spectrum of compound **4a**

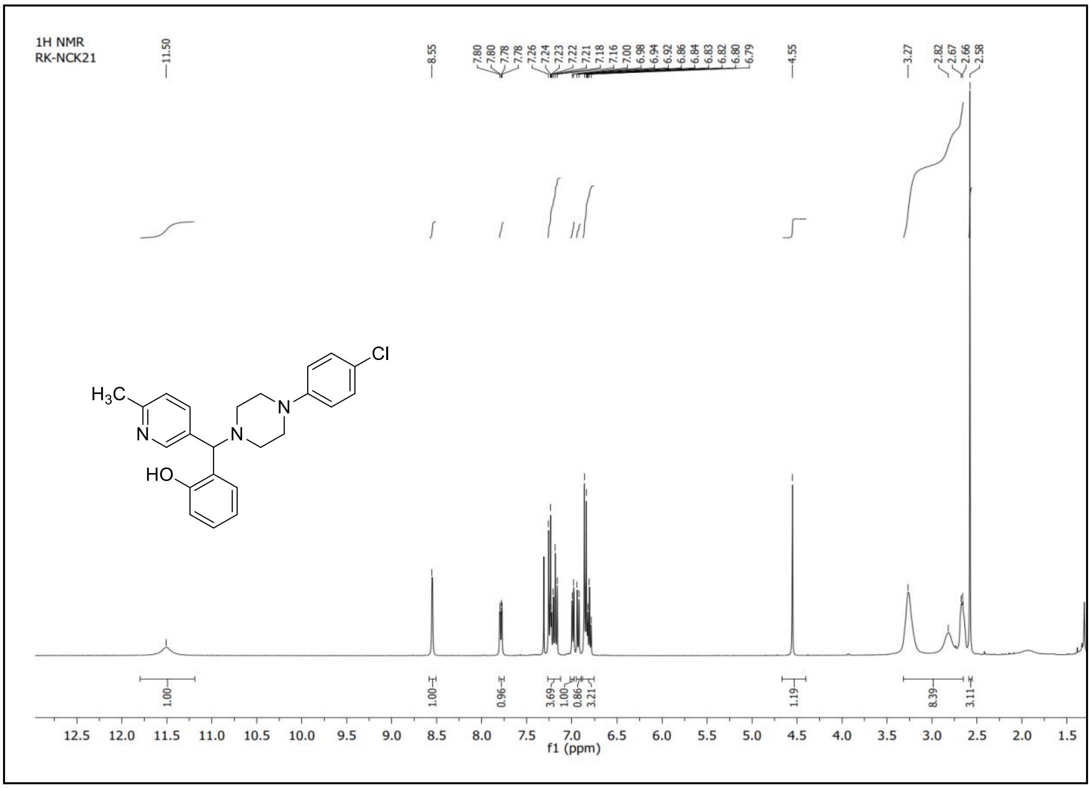


S3:$^{1}$H NMR spectrum of compound **4b**

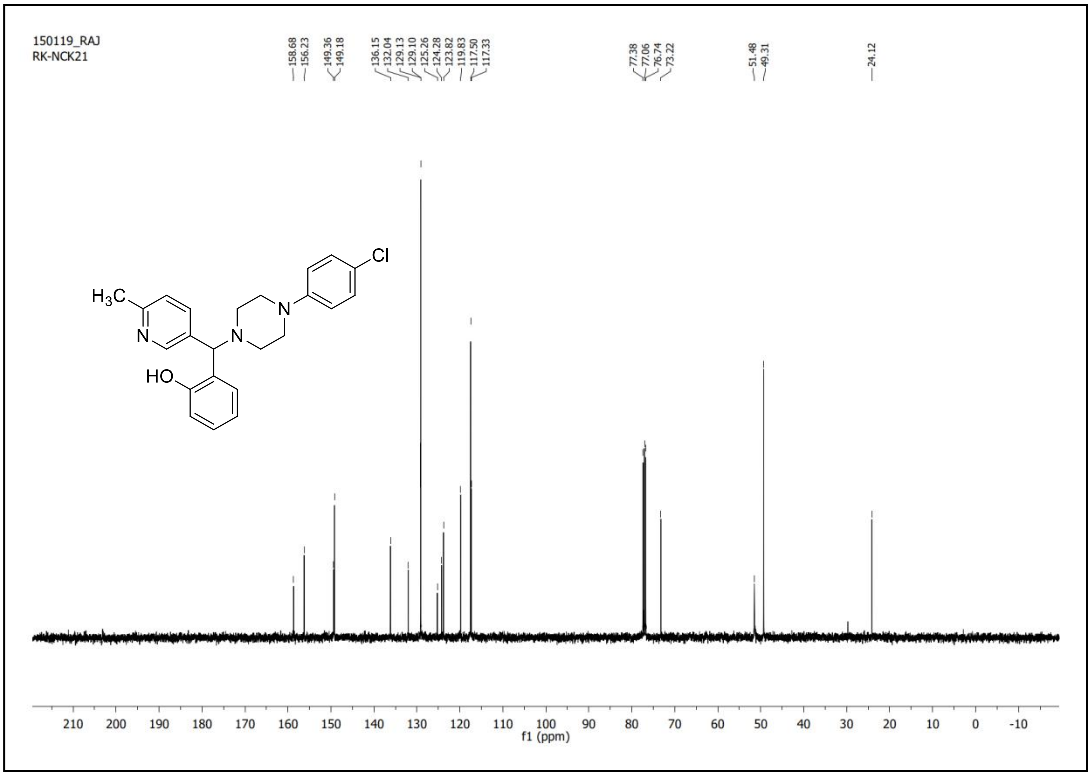


S4:[13] C NMR spectrum of compound **4b**

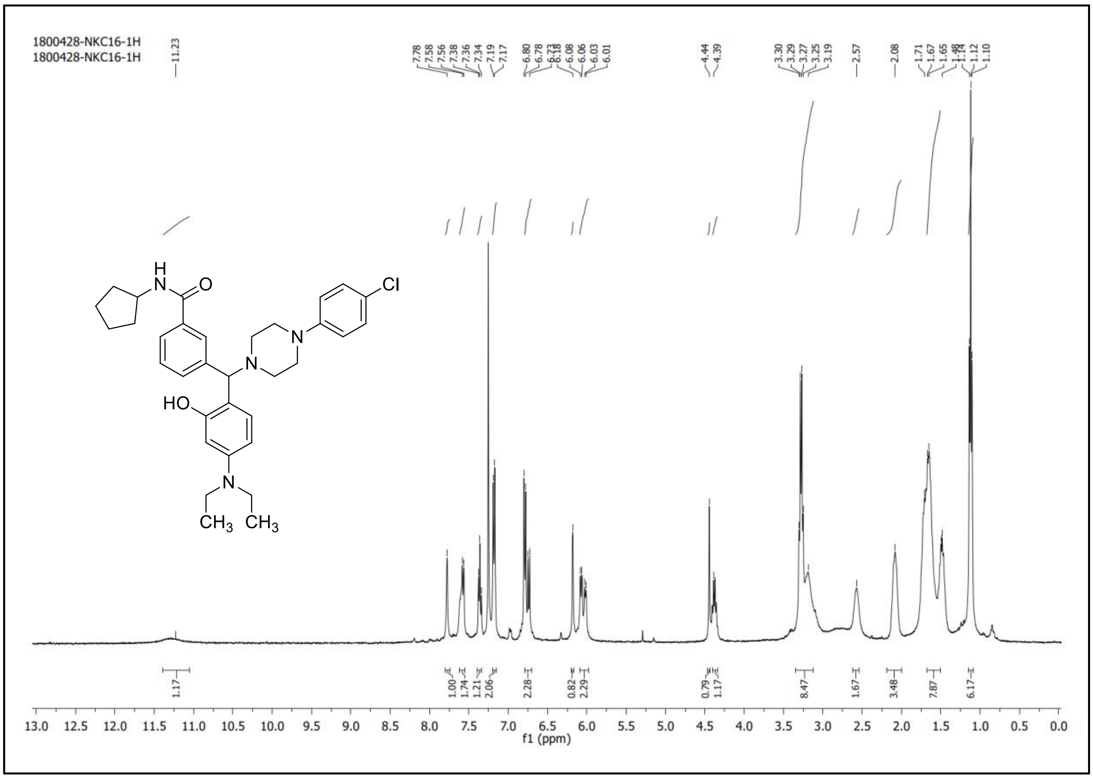


S5:$^{1}$H NMR spectrum of compound **4c**

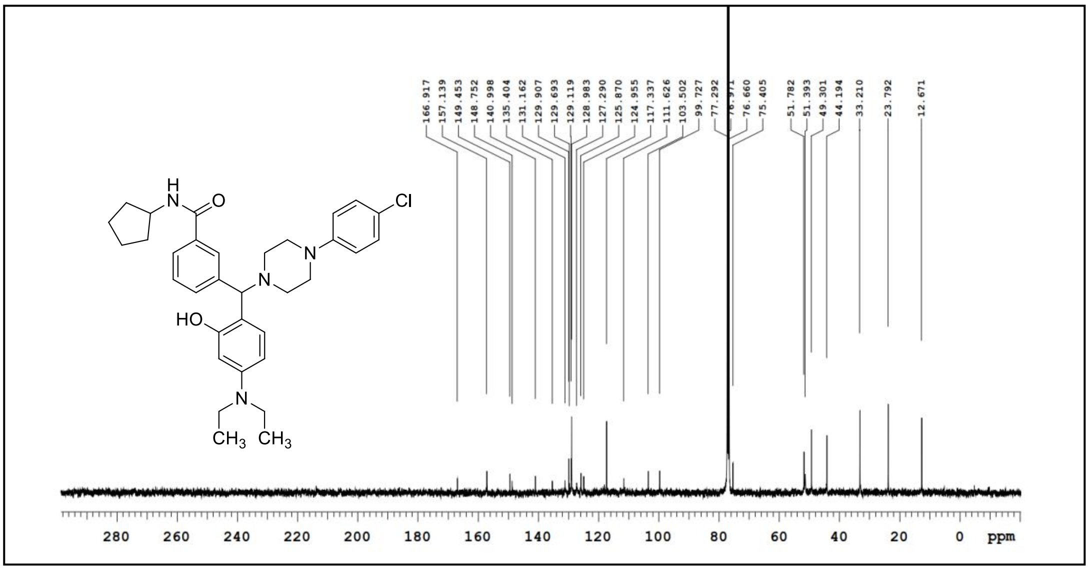


S6:$^{13}$C NMR spectrum of compound **4c**

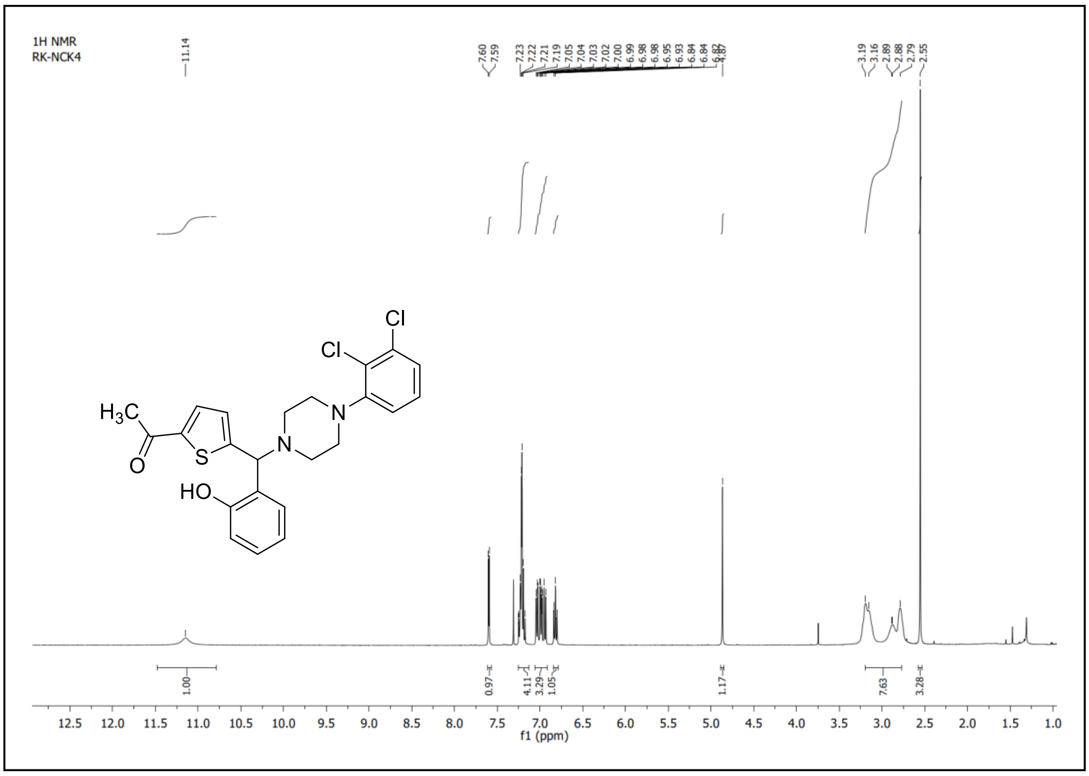


S7:$^{1}$H NMR spectrum of compound **4d**

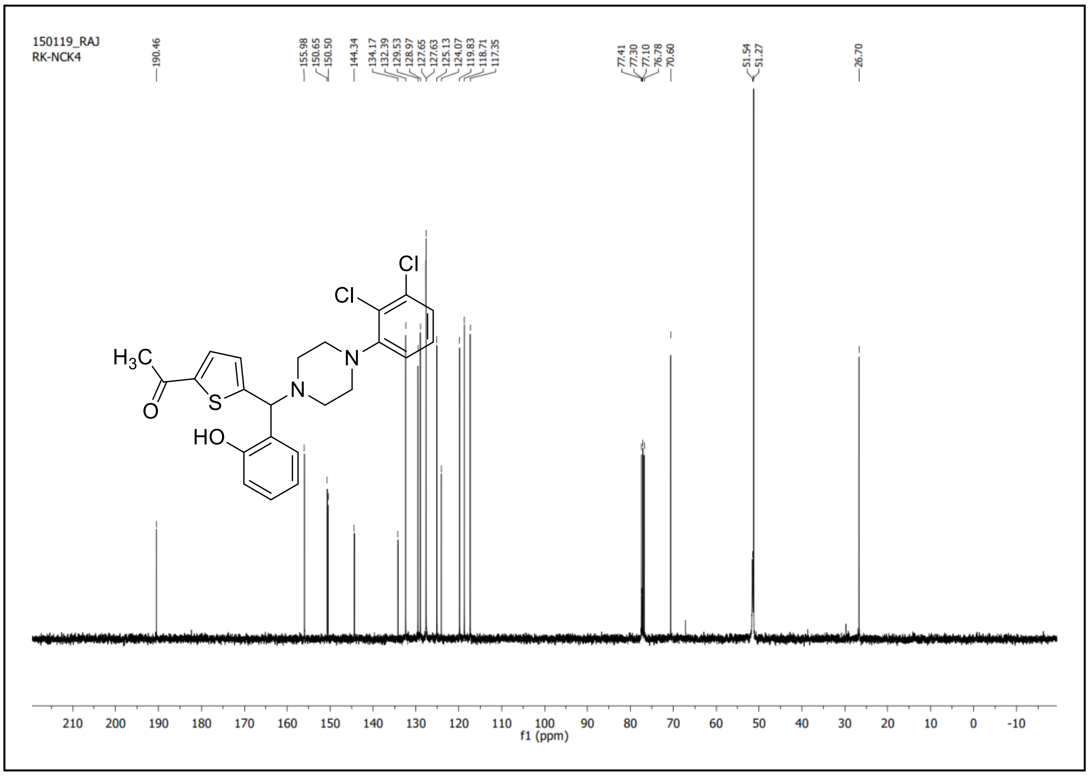


S8:$^{13}$C NMR spectrum of compound **4d**

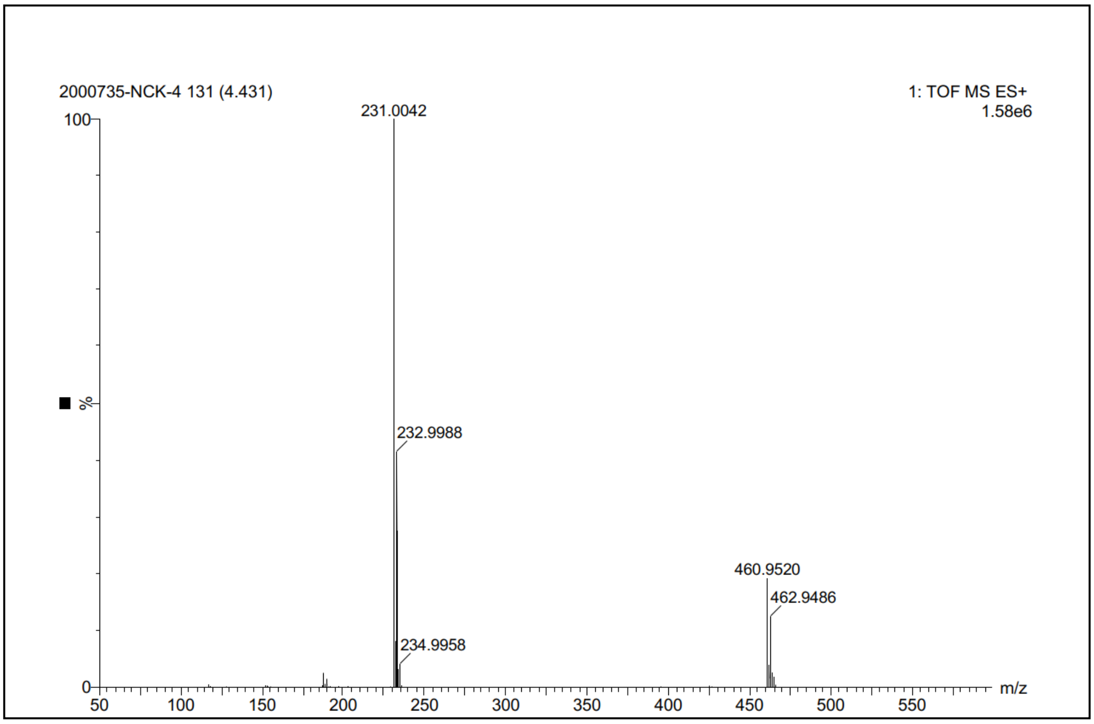


S9:Mass spectrum of compound **4d**

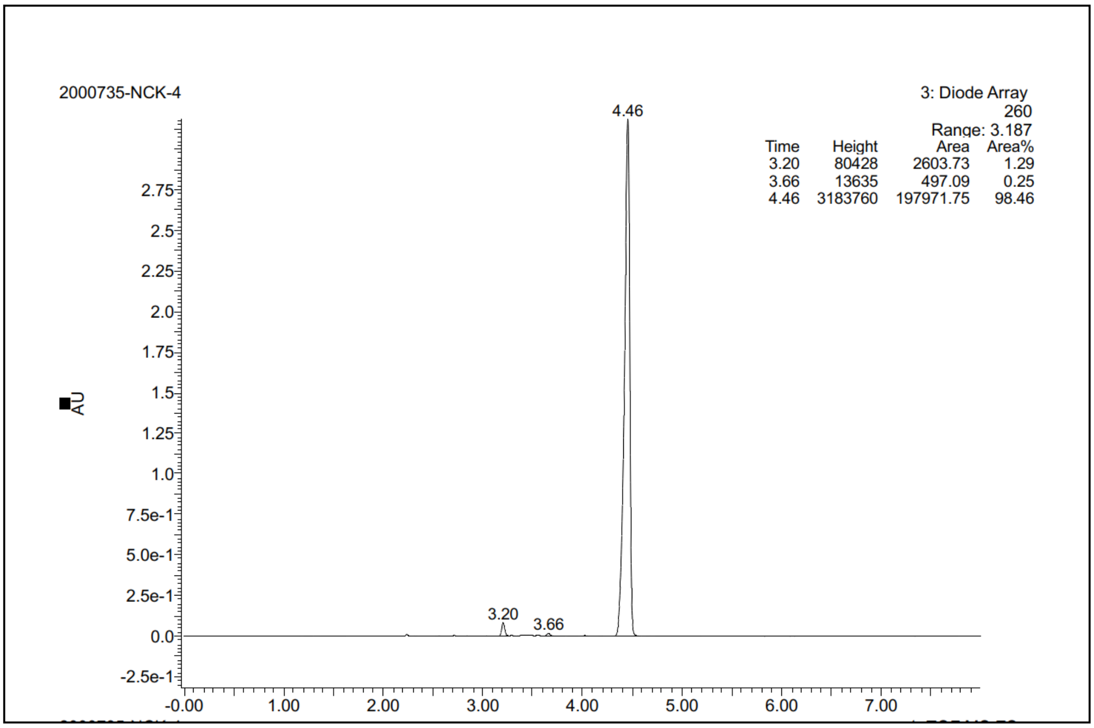


S10:LC chromatogram of compound **4d**

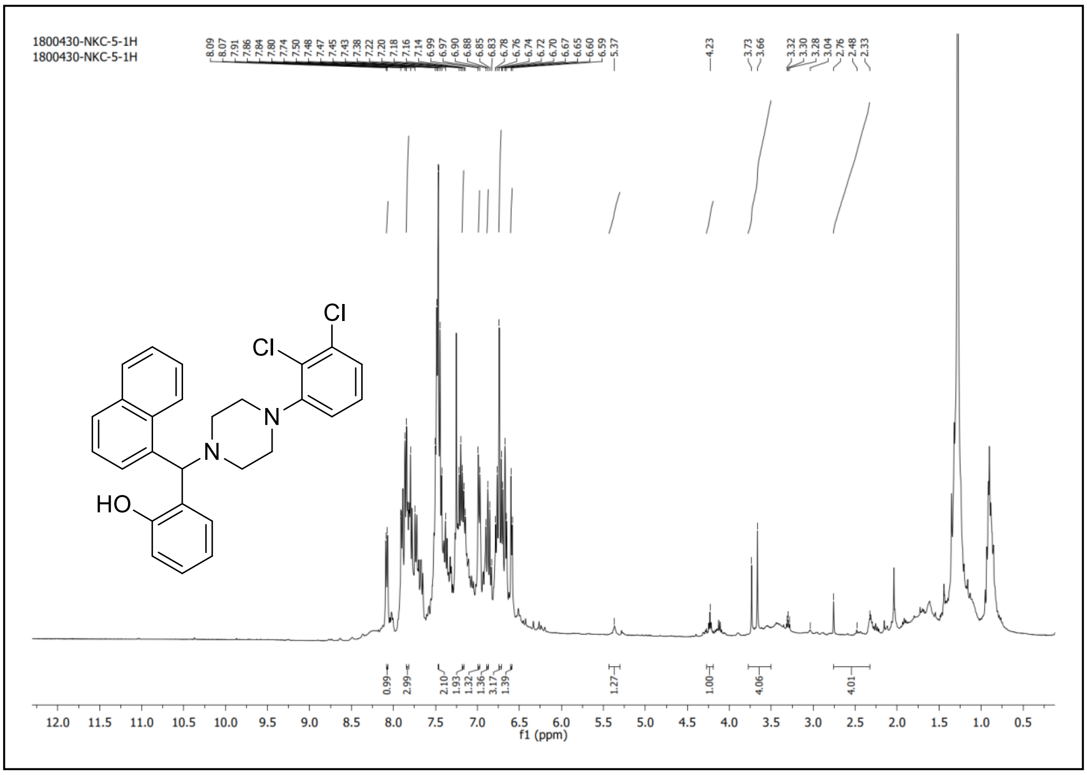


S11:$^{1}$H NMR spectrum of compound **4e**

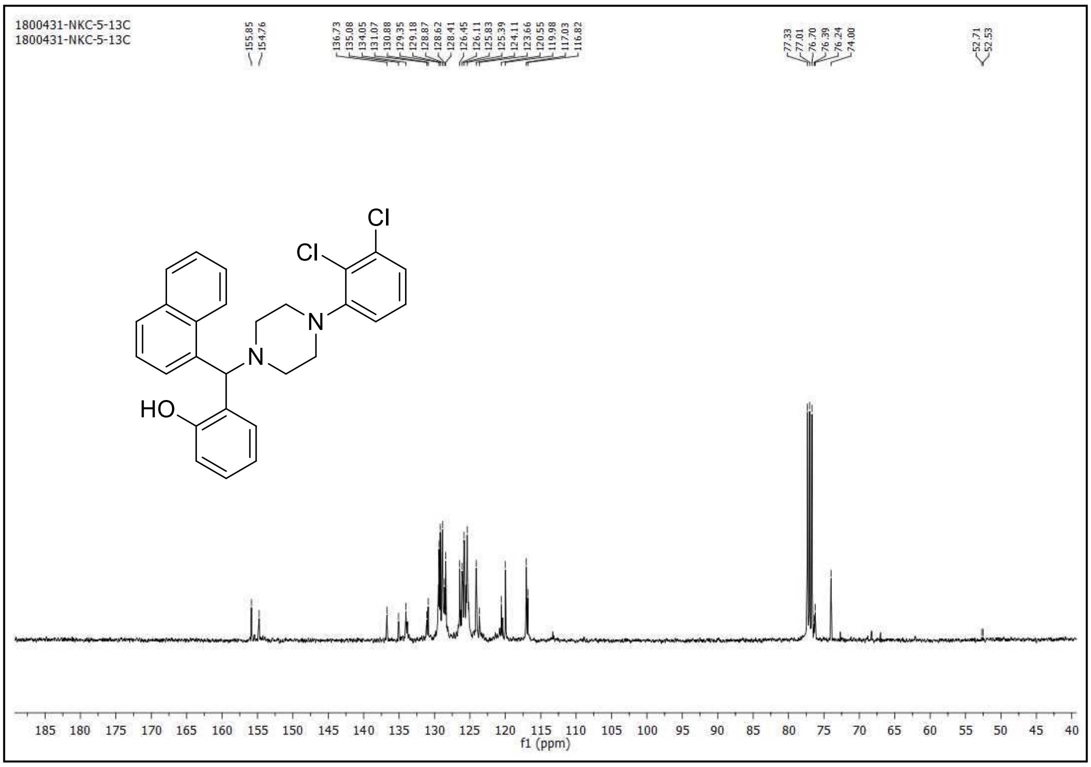


S12:$^{13}$C NMR spectrum of compound **4e**

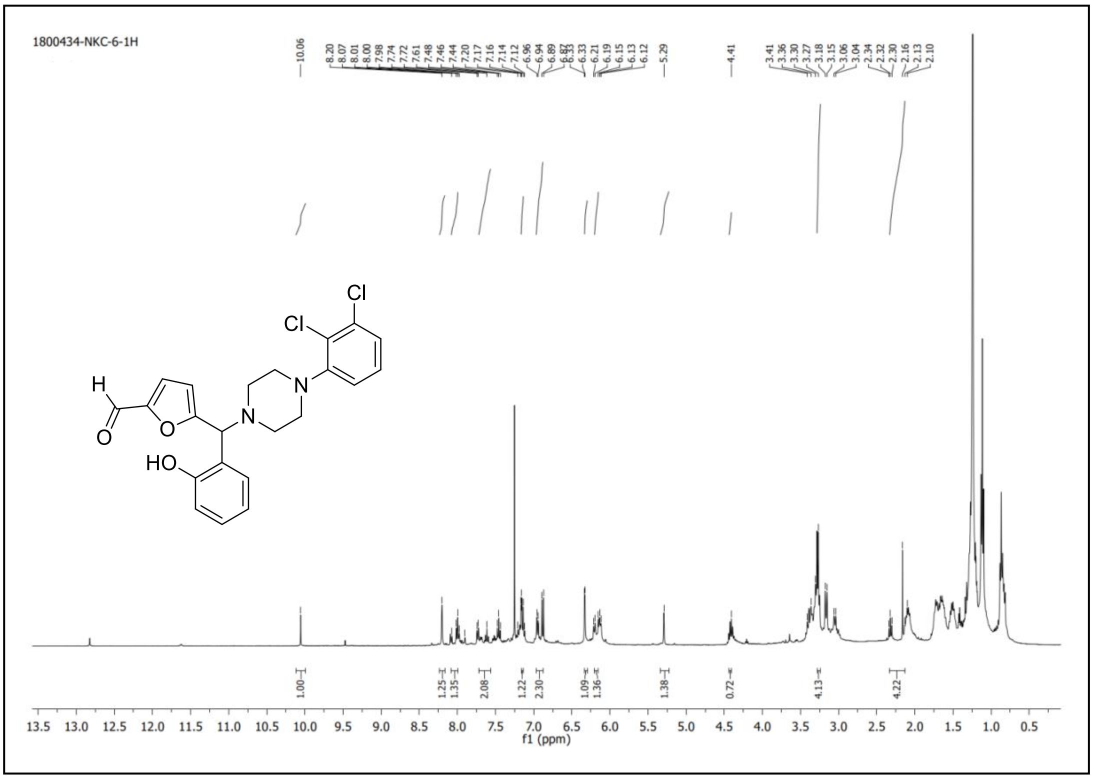

S13:$^{1}$H NMR spectrum of compound **4f**

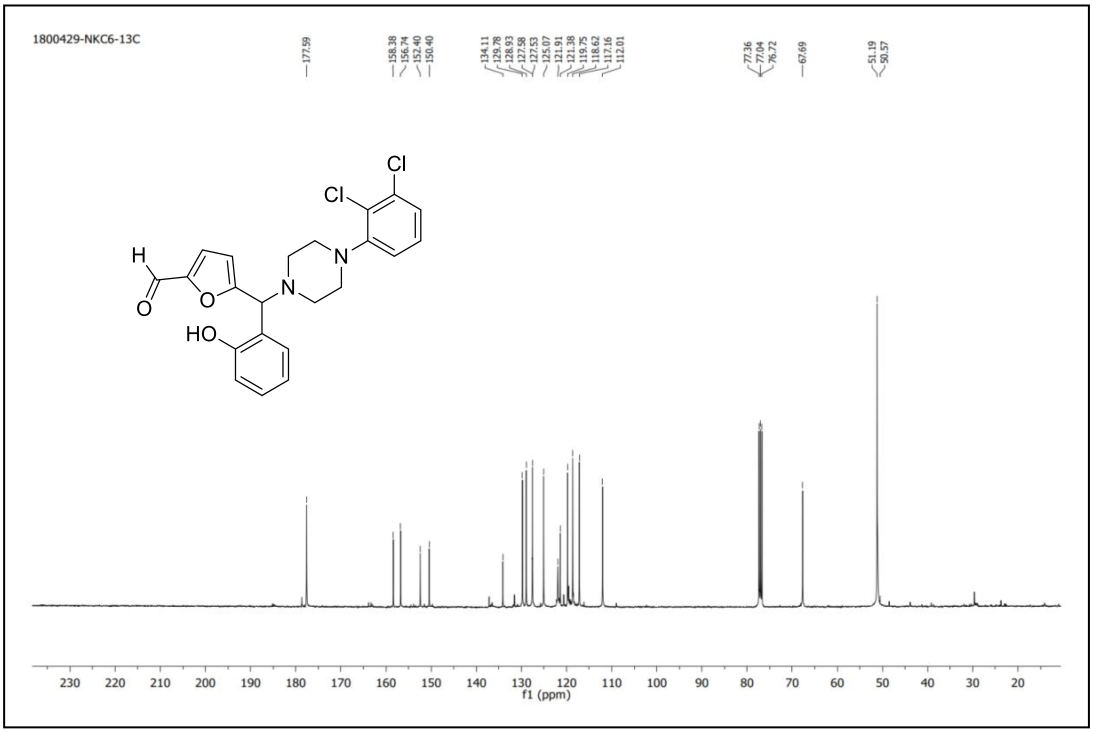

S14:$^{13}$C NMR spectrum of compound **4f**

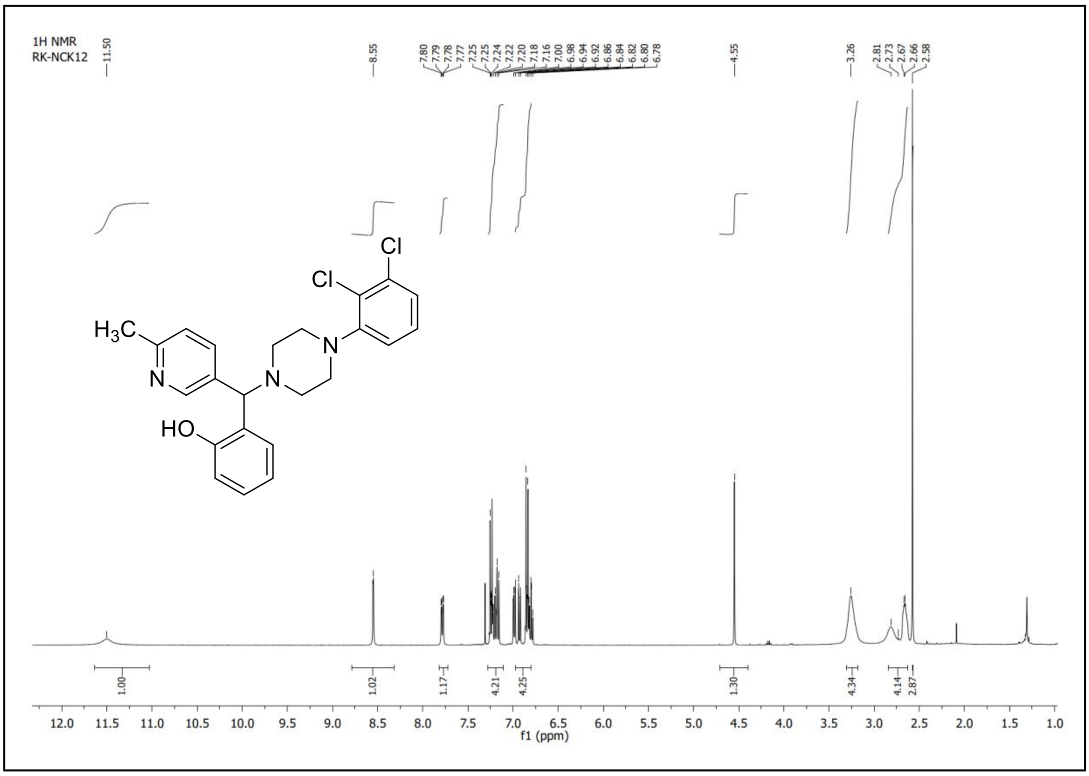


S15:$^{1}$H NMR spectrum of compound **4g**

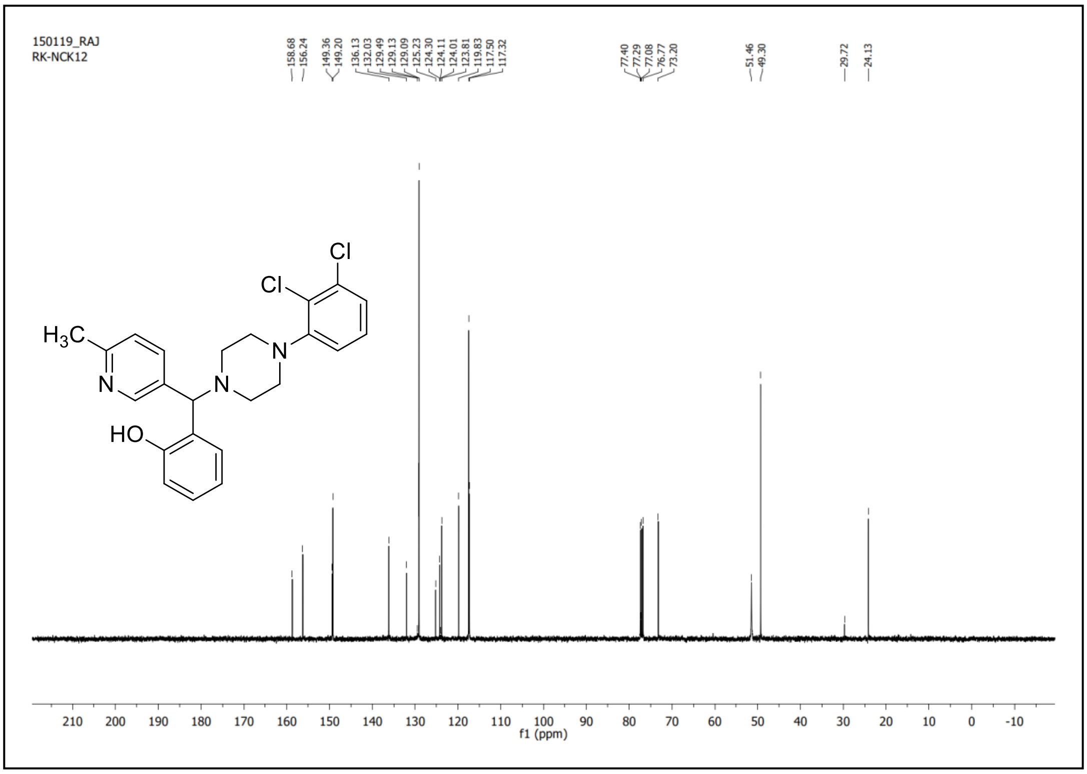


S16:$^{13}$ C NMR spectrums of compound **4g**

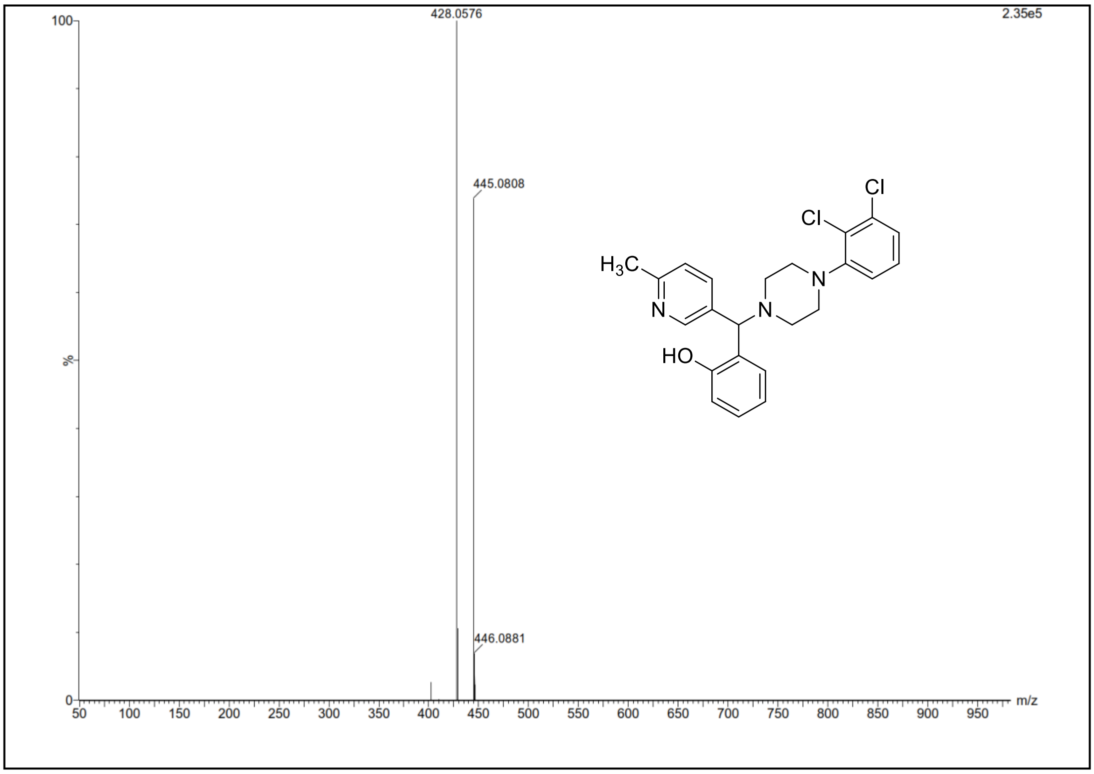


S17:Mass spectrum of compound **4g**

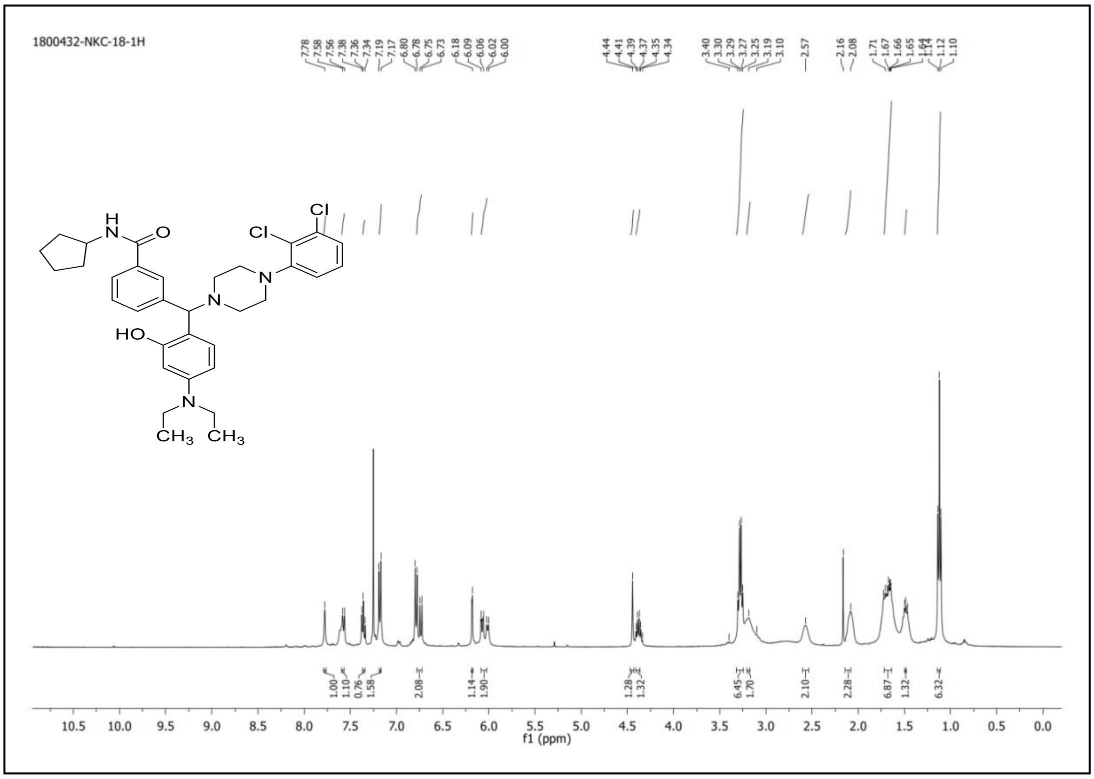


S18:$^{1}$H NMR spectrum of compound **4h**

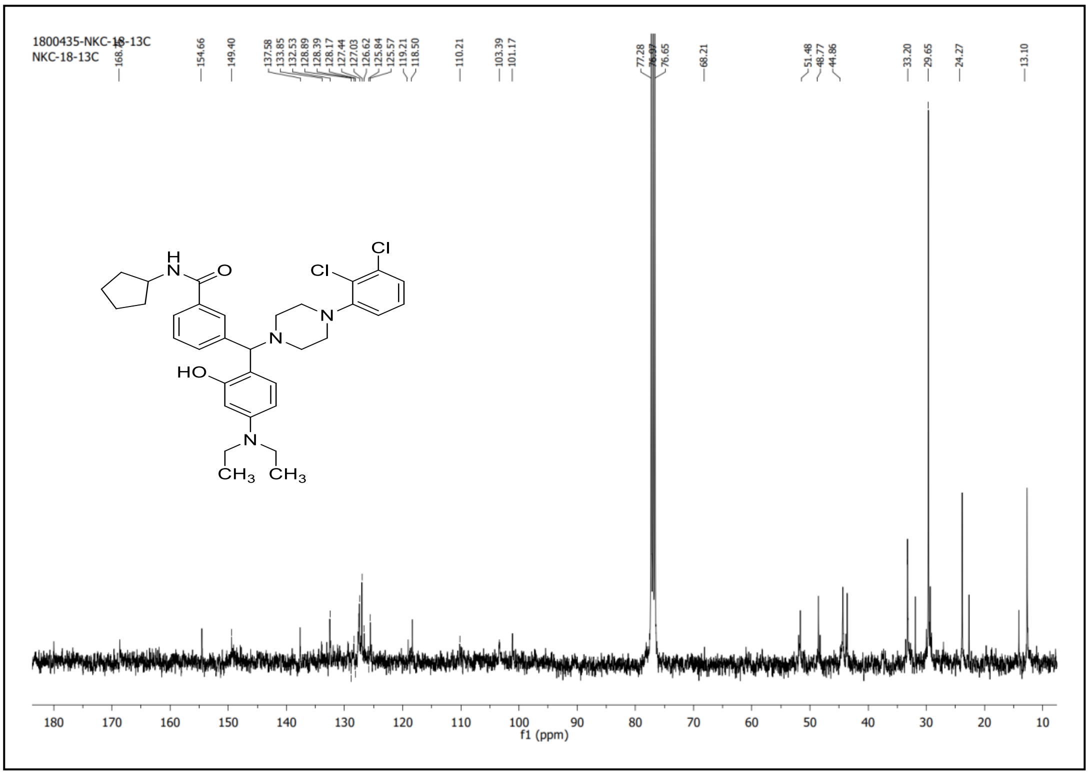

S19:$^{13}$ C NMR Spectrum of compound **4h**

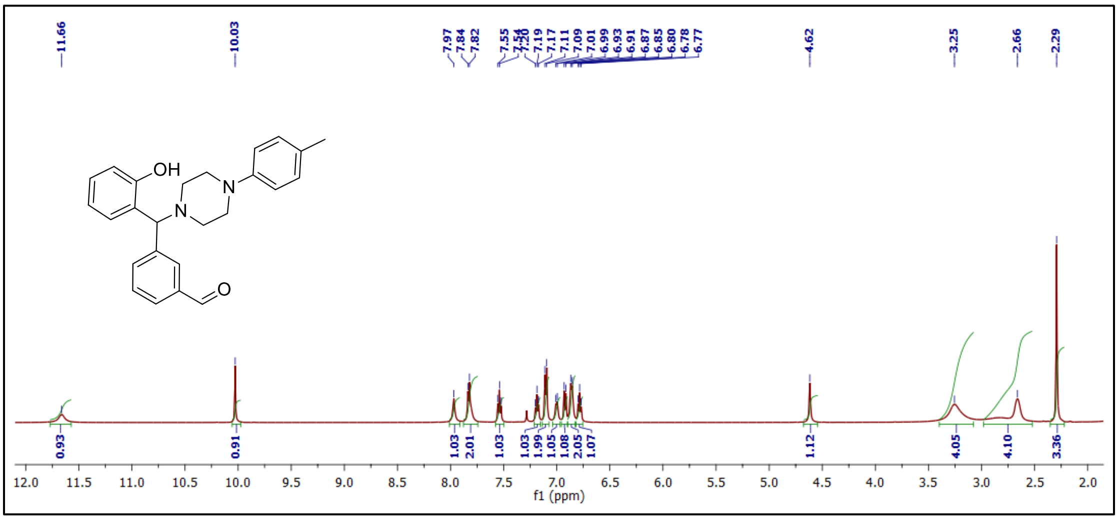

Sp20:$^{1}$H NMR Spectrum of compound **4i**

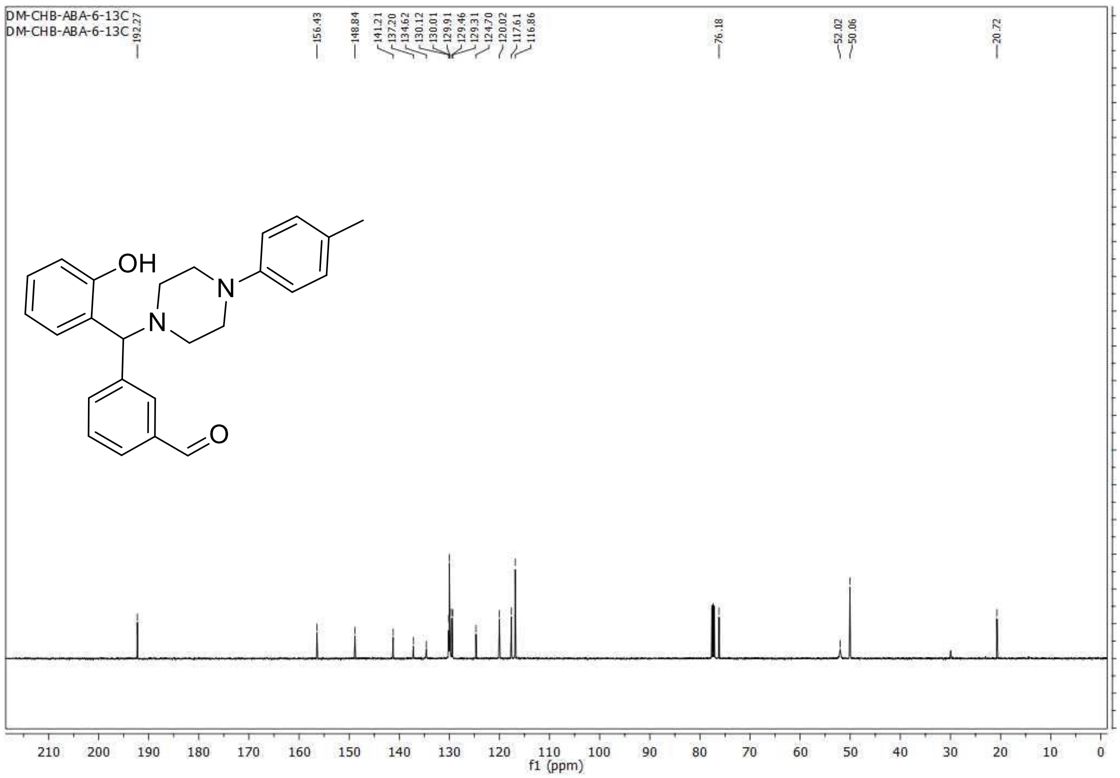


S21:$^{13}$C NMR Spectrum of compound **4i**

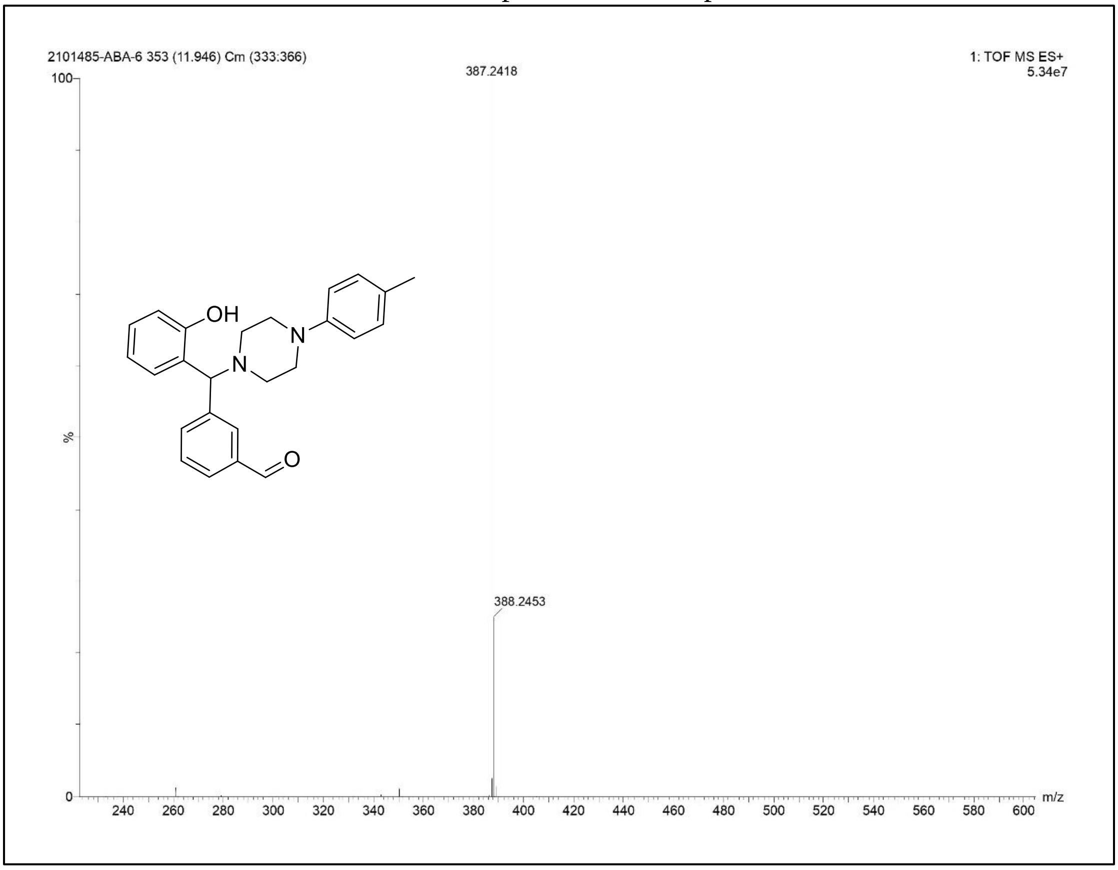


S22: Mass spectrum of compound **4i**

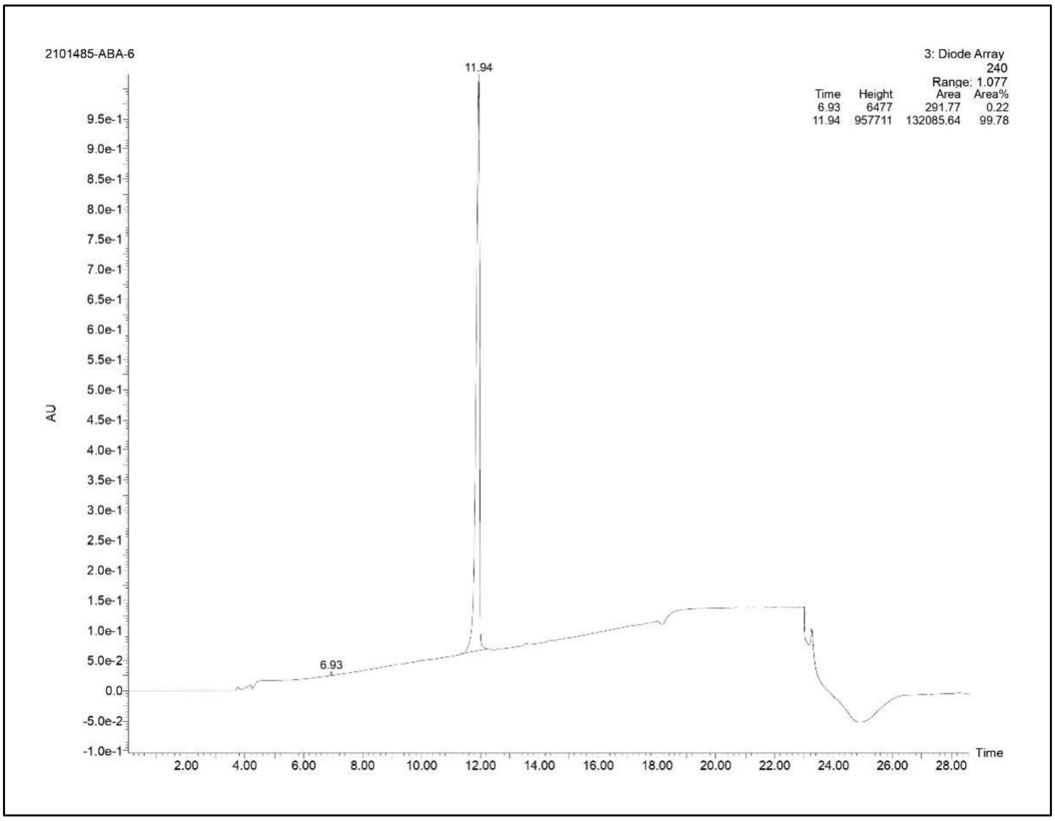


S23: HPLC Spectrum of compound **4i**

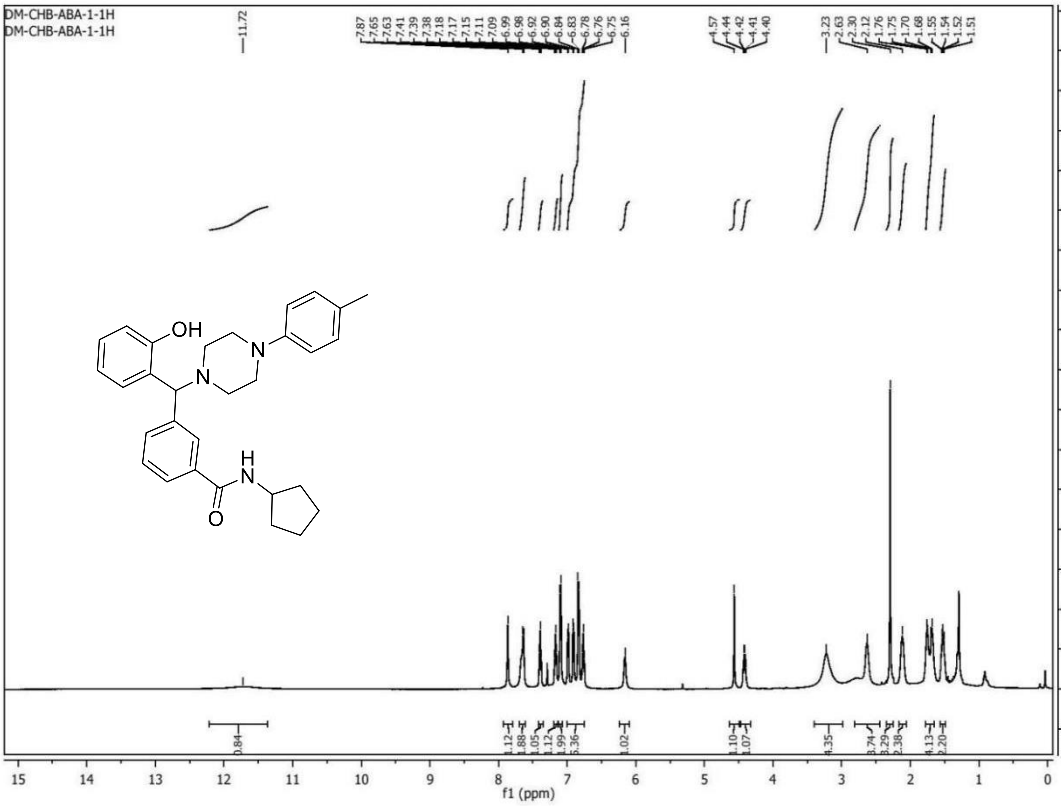


S24:$^{1}$H NMR Spectrum of compound **4j**

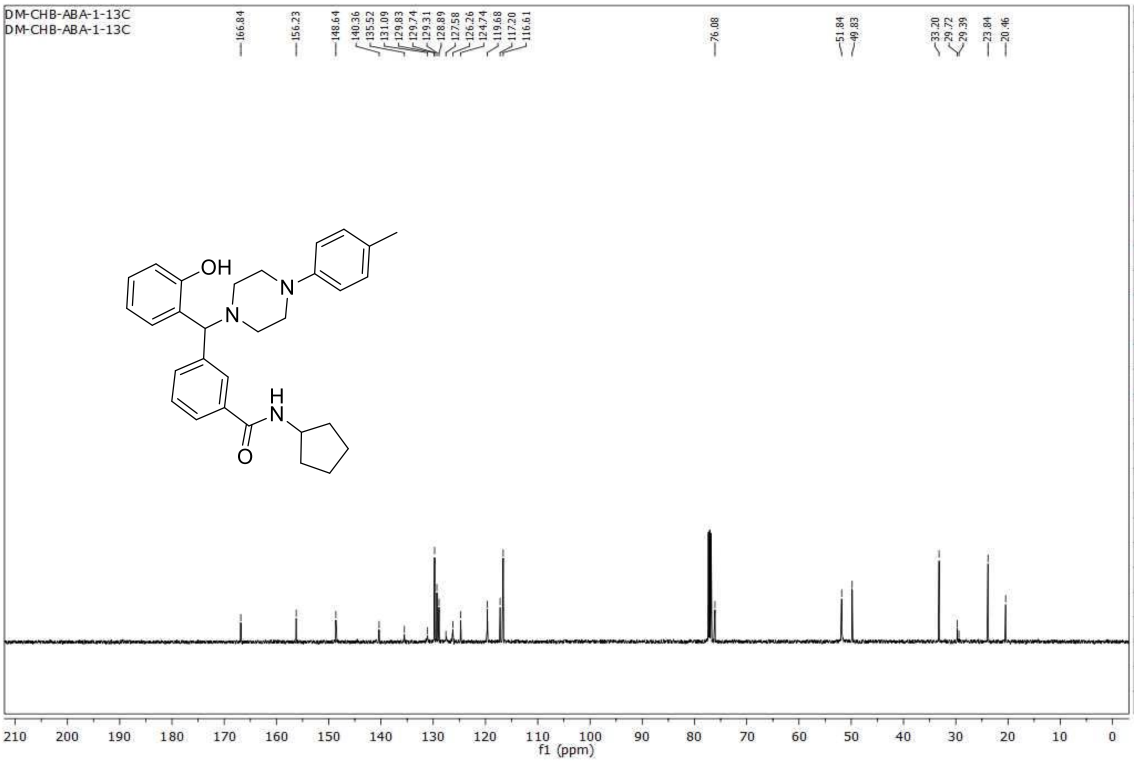


S25:$^{13}$C NMR Spectrum of compound **4j**

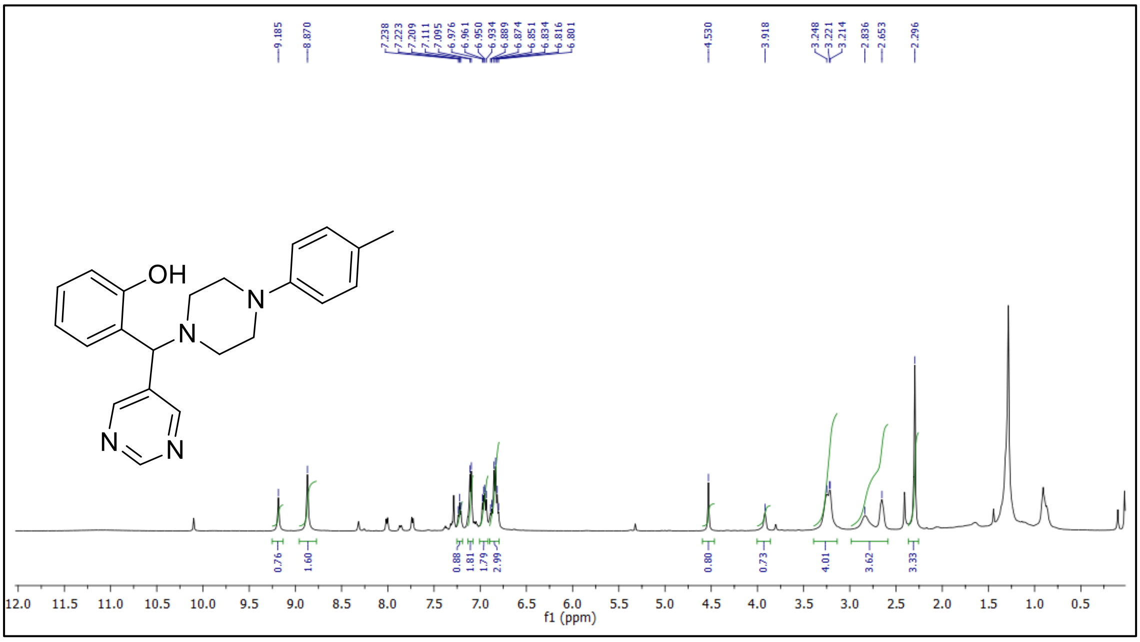


S26:$^{1}$H NMR Spectrum of compound **4k**

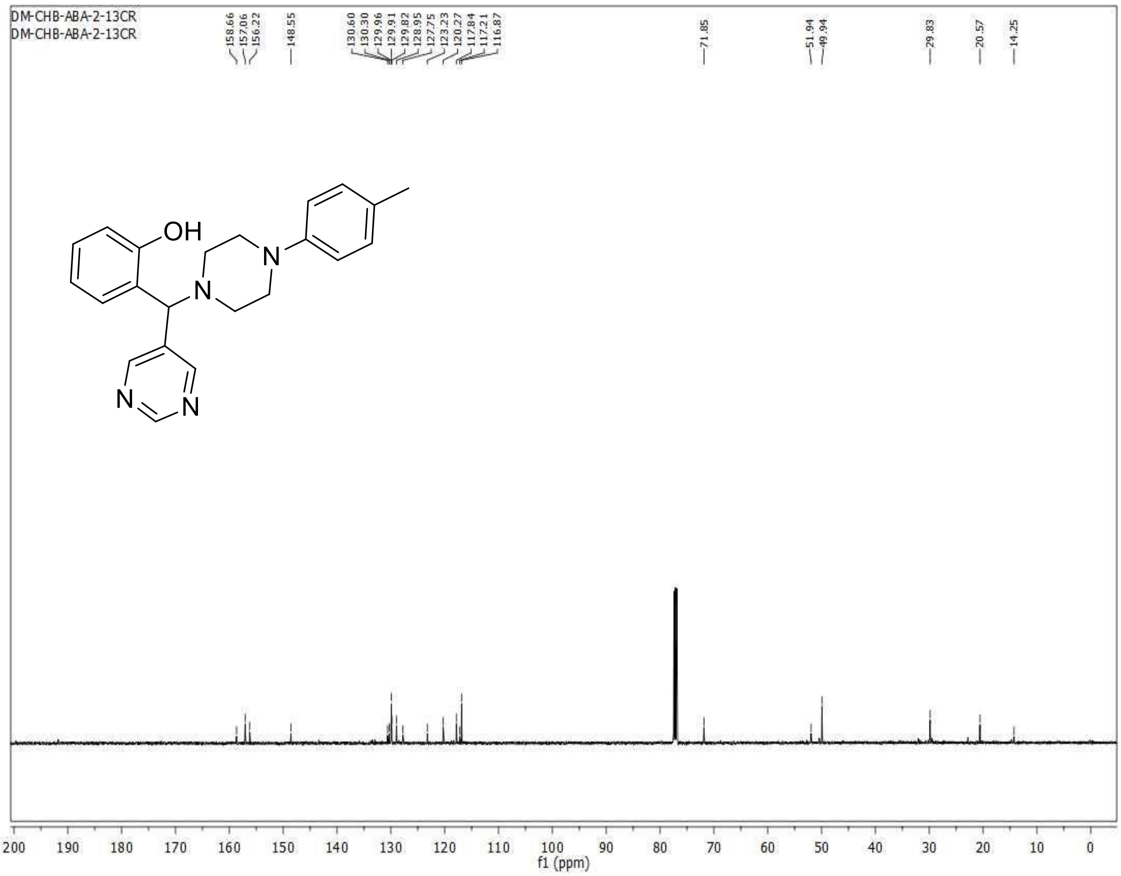

S27:$^{13}$C NMR Spectrum of compound **4k**

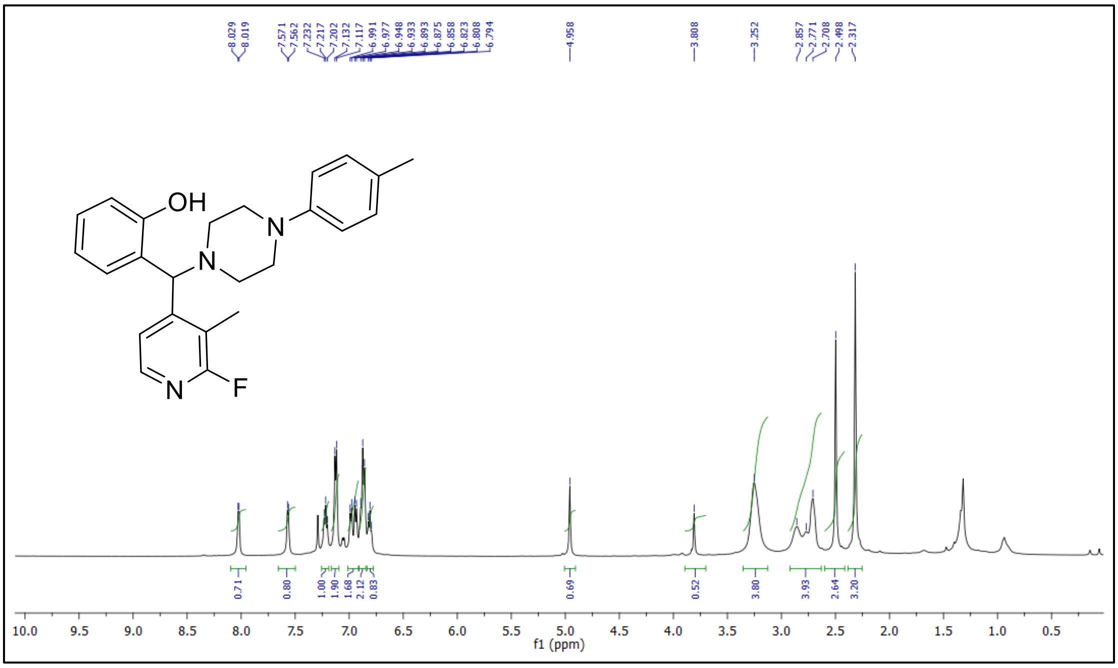

S28:$^{1}$H NMR Spectrum of compound **4l.**

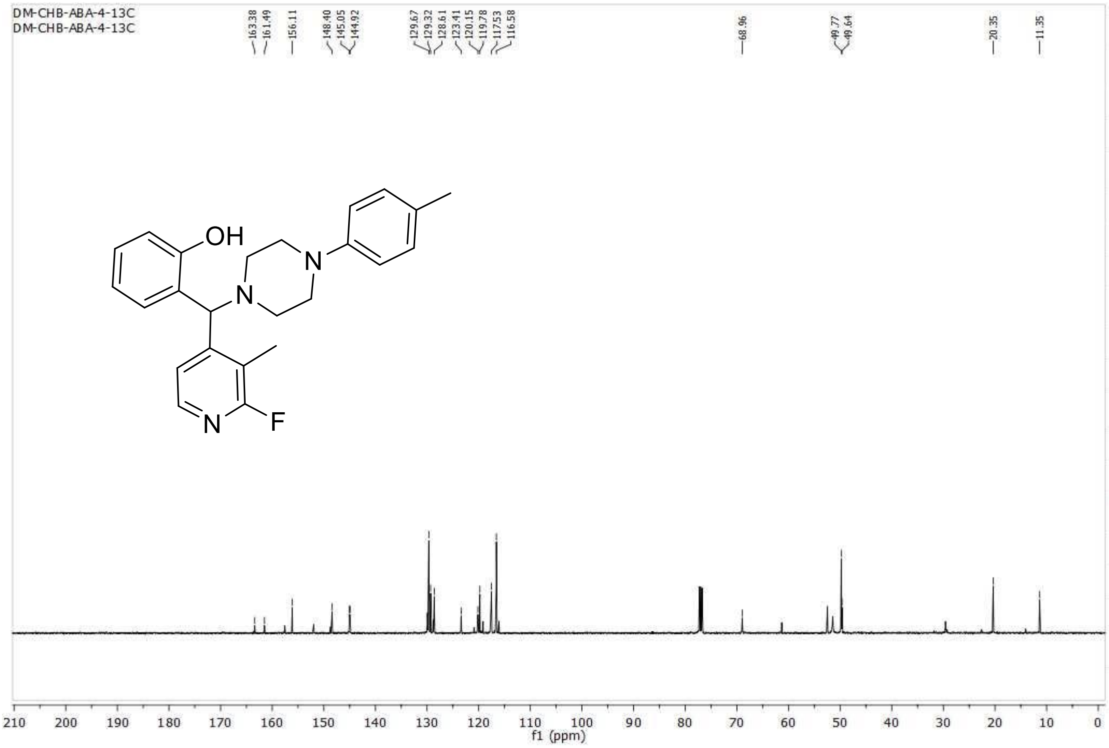


S29:$^{13}$C NMR Spectrum of compound **4l.**

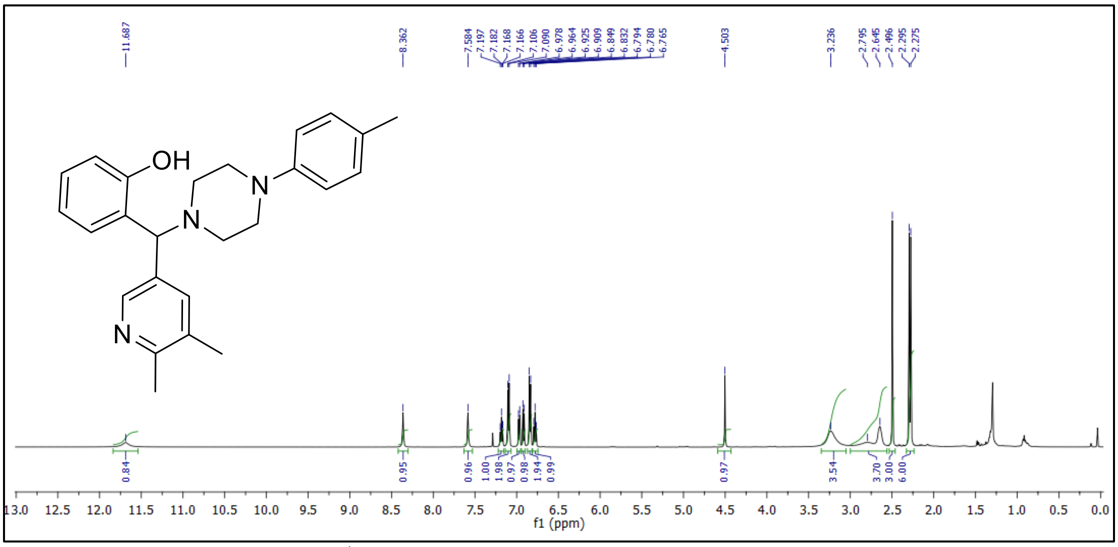


S30:$^{1}$H NMR Spectrum of compound **4m.**

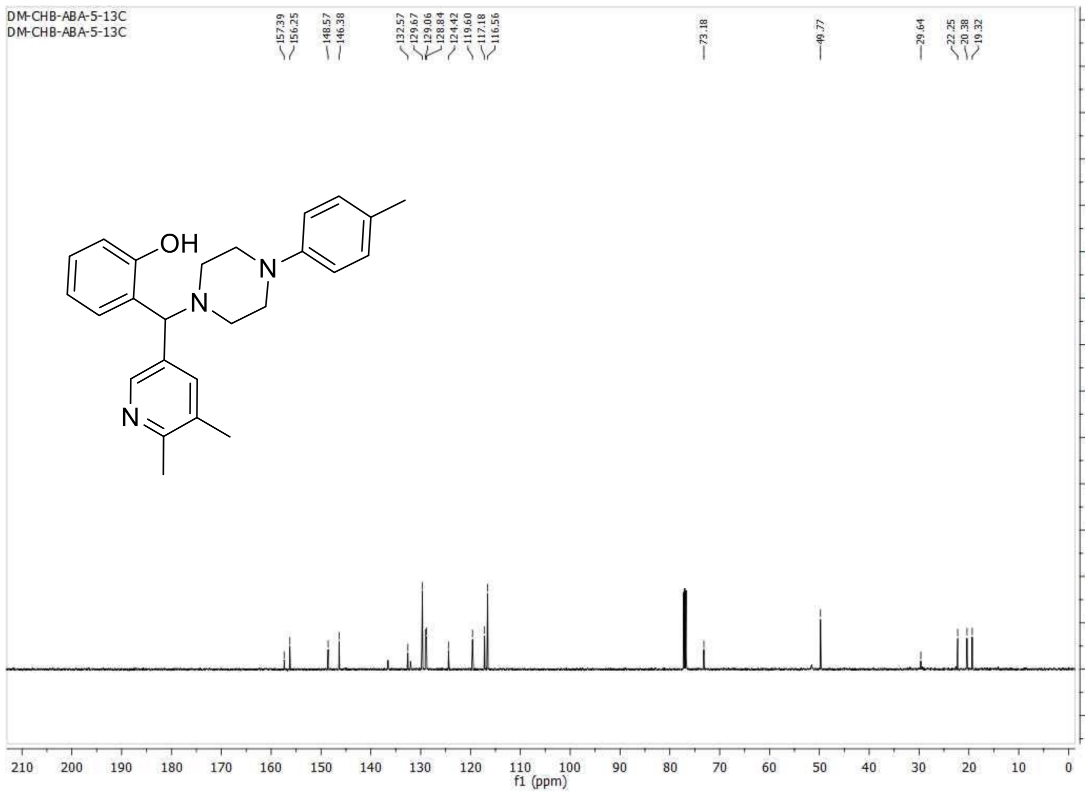

S31:$^{13}$C NMR Spectrum of compound **4m**

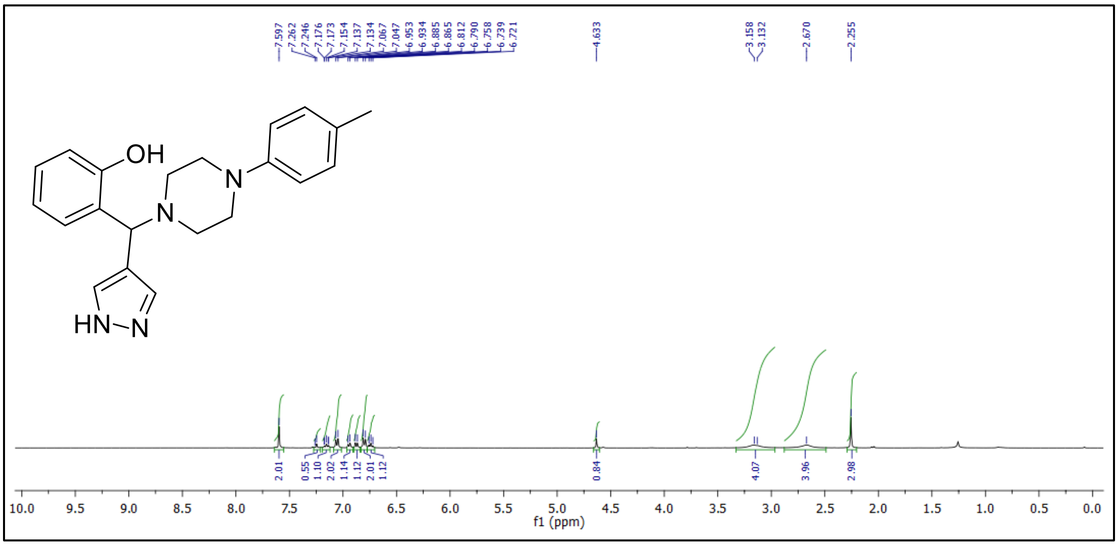

S32:$^{1}$H NMR Spectrum of compound **4n**

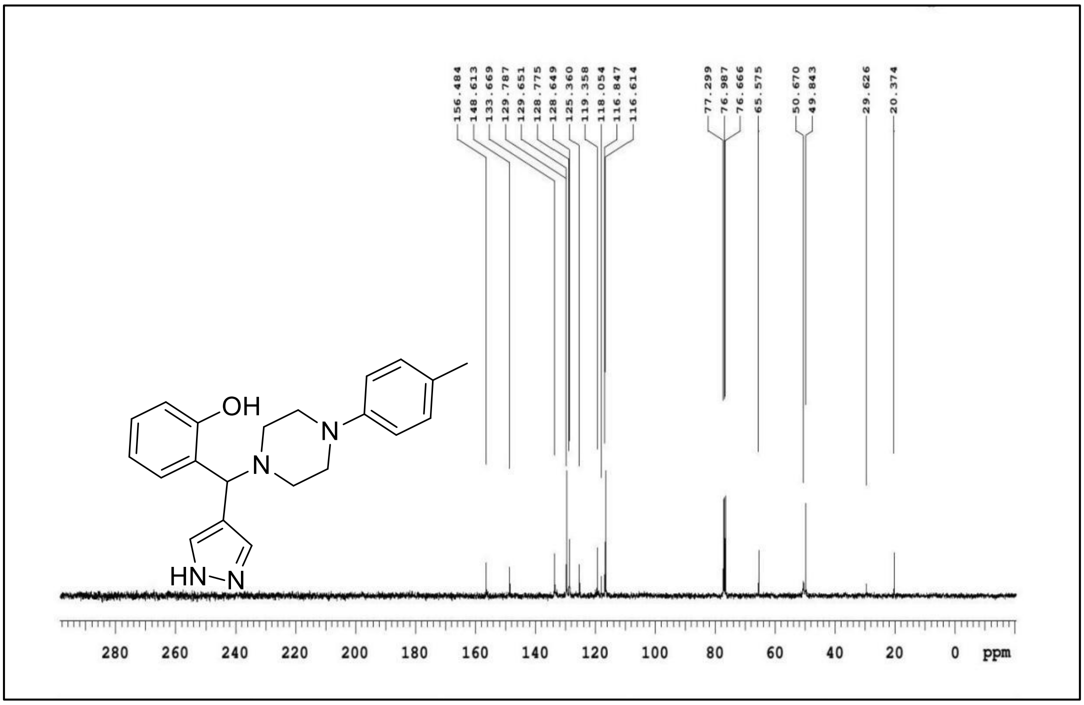


S33:$^{13}$C NMR Spectrum of compound **4n**

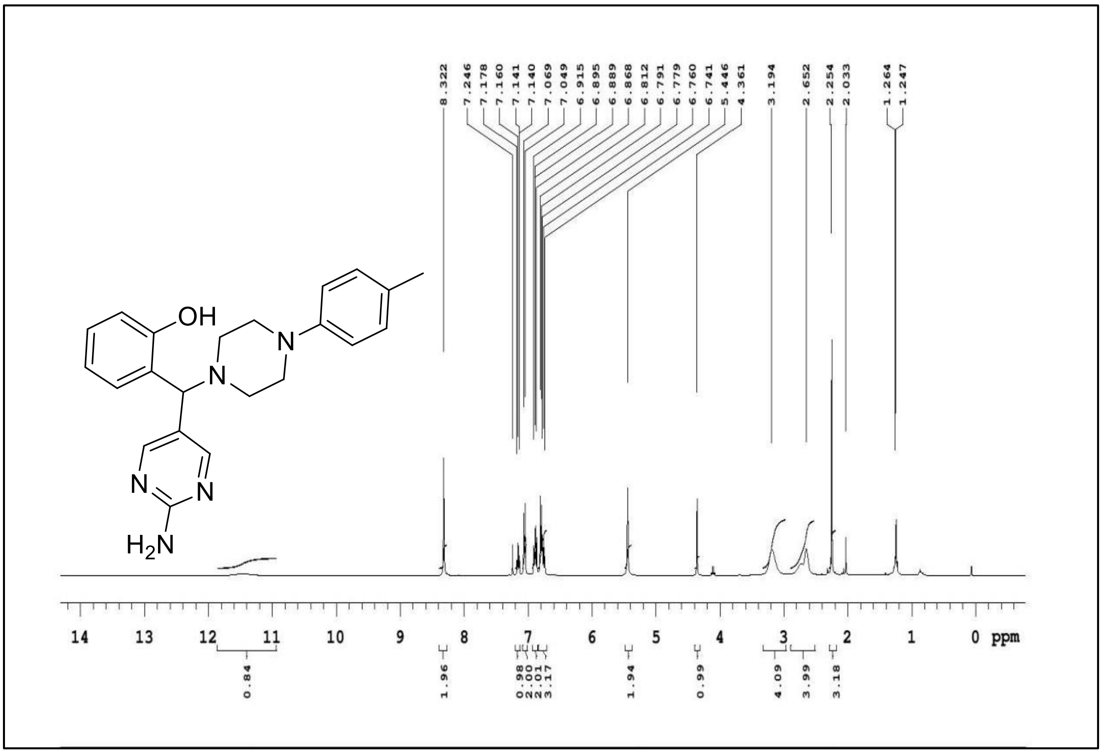


S34:$^{1}$H NMR Spectrum of compound **4o**

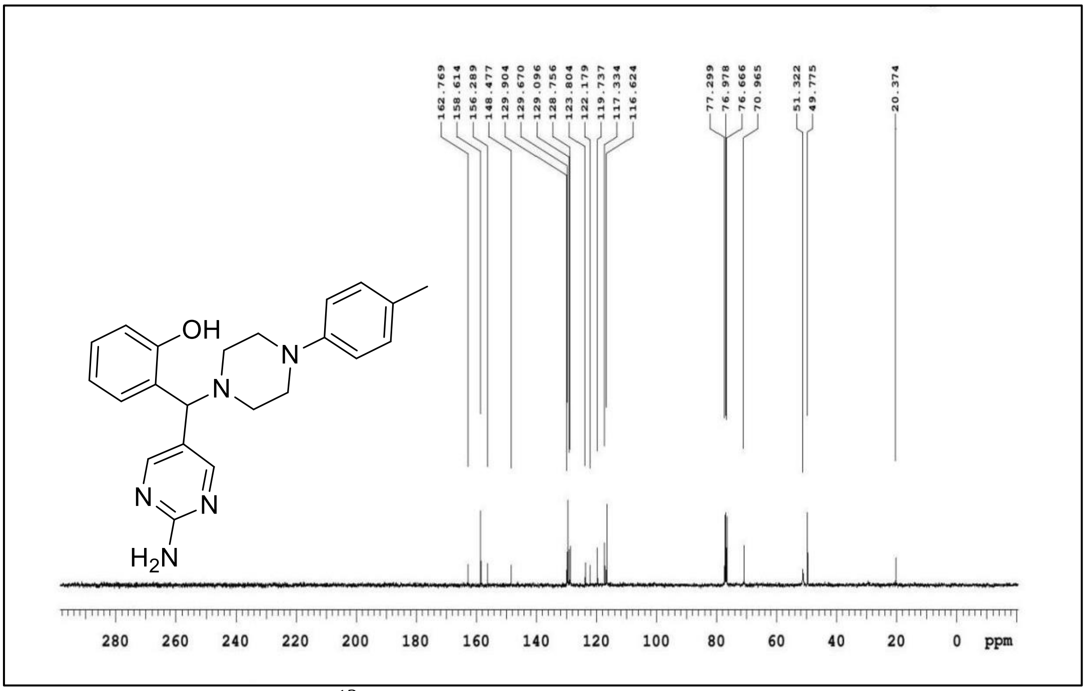


S35:$^{13}$C NMR Spectrum of compound **4o**

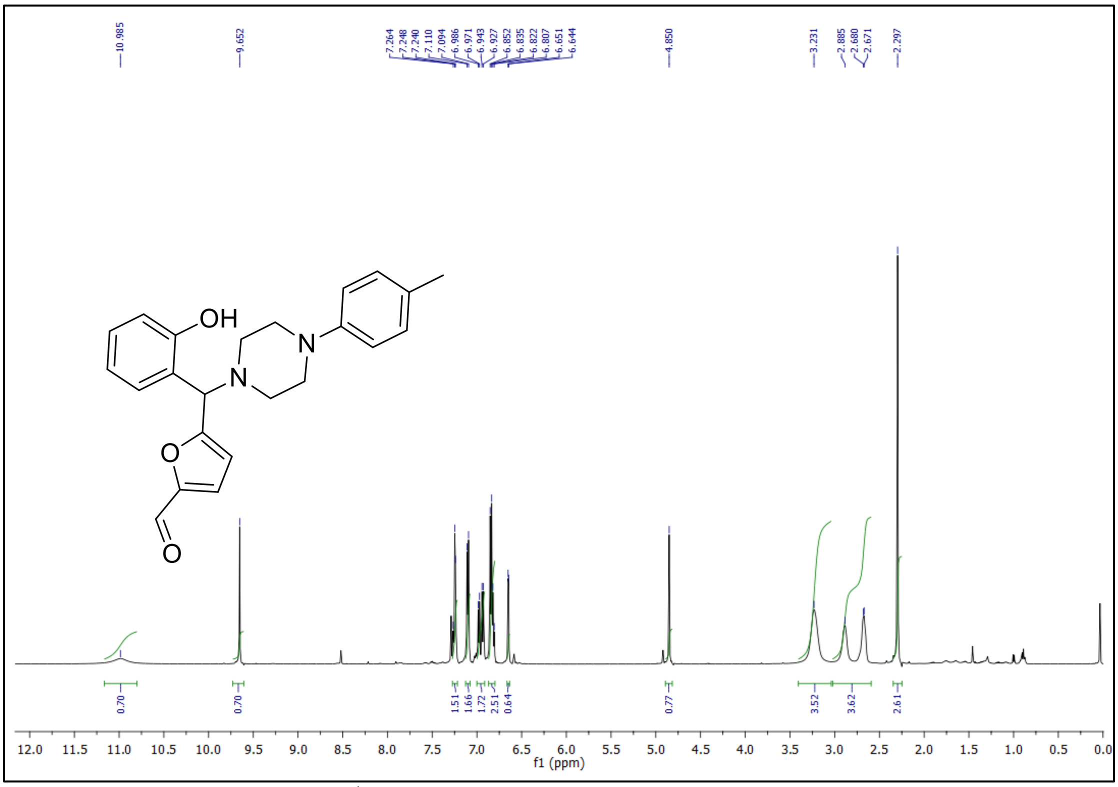


S36:$^{1}$H NMR Spectrum of compound **4p**

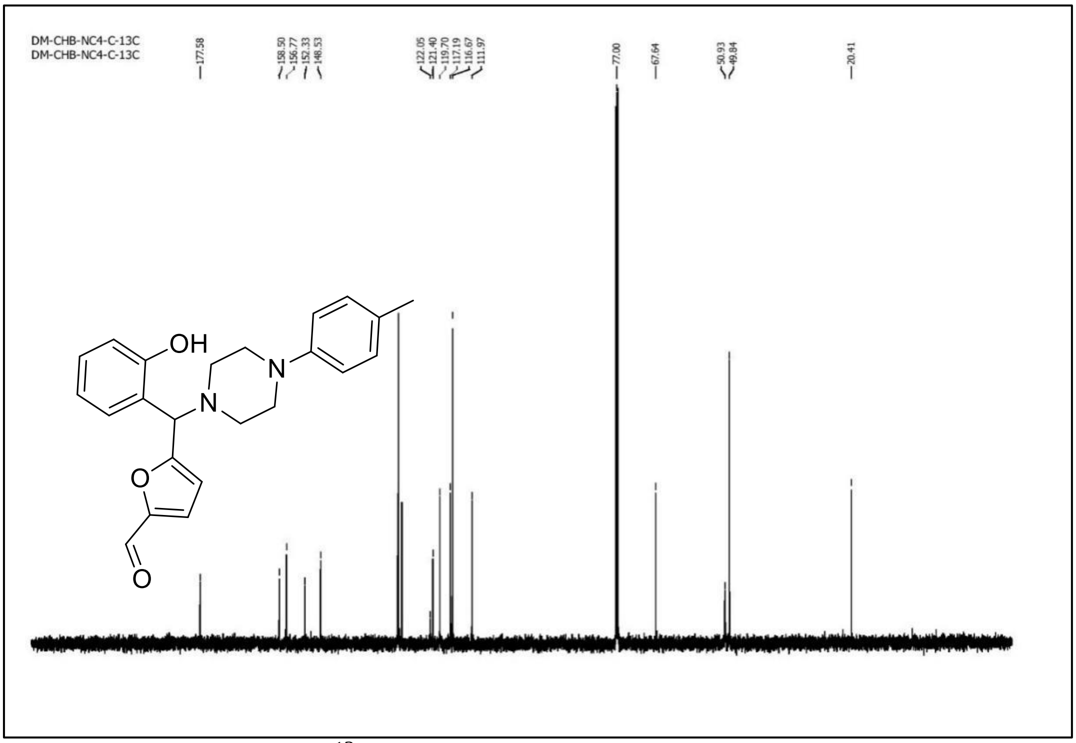


S37: $^{13}$C NMR Spectrum of compound **4p**

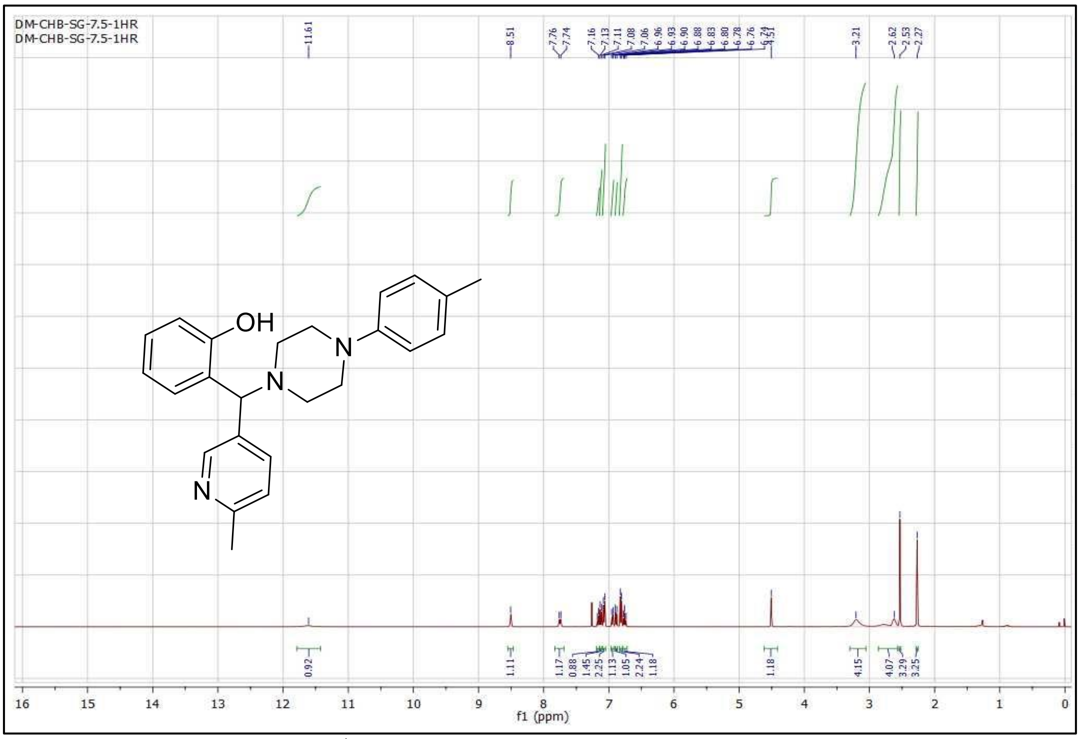


S38: $^{1}$H NMR Spectrum of compound **4q**

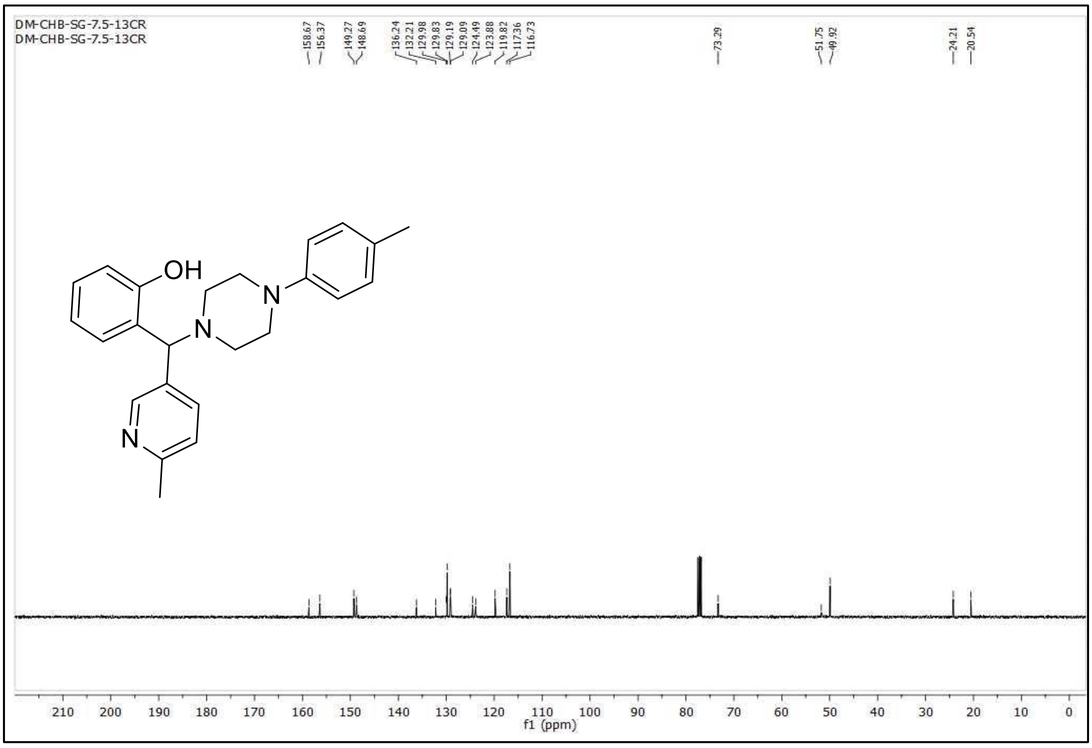


S39:$^{13}$C NMR Spectrum of compound **4q**

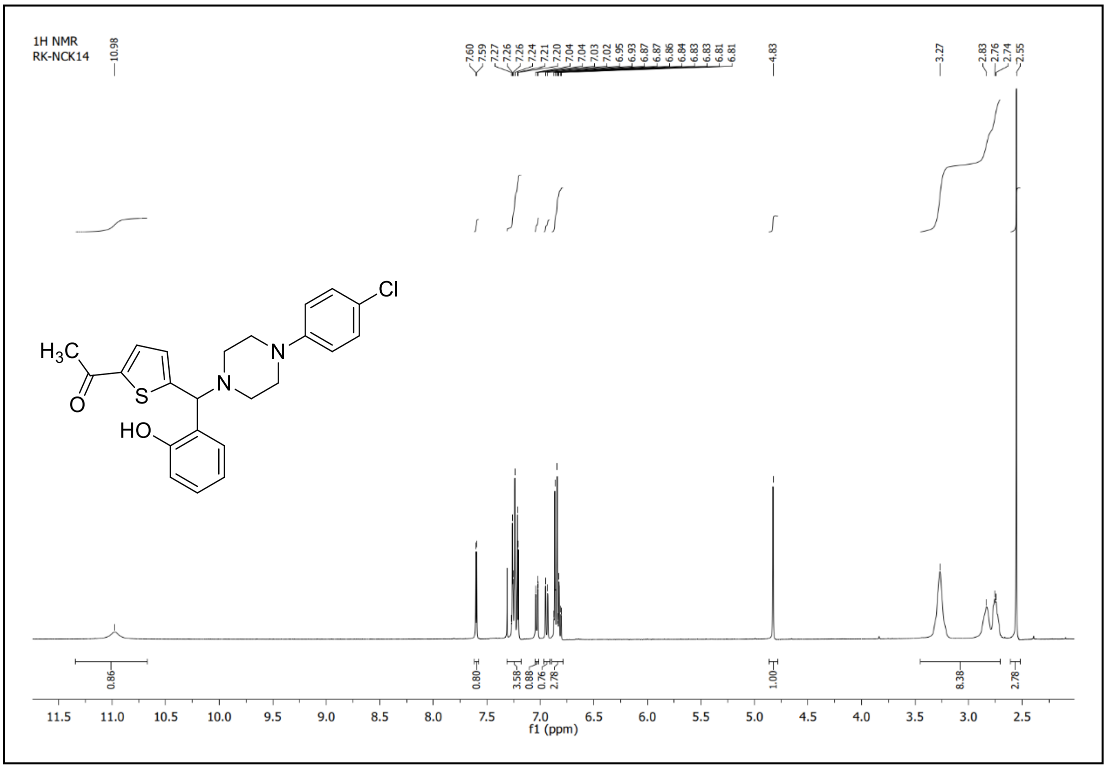


S40:$^1$H NMR Spectrum of compound **4r**

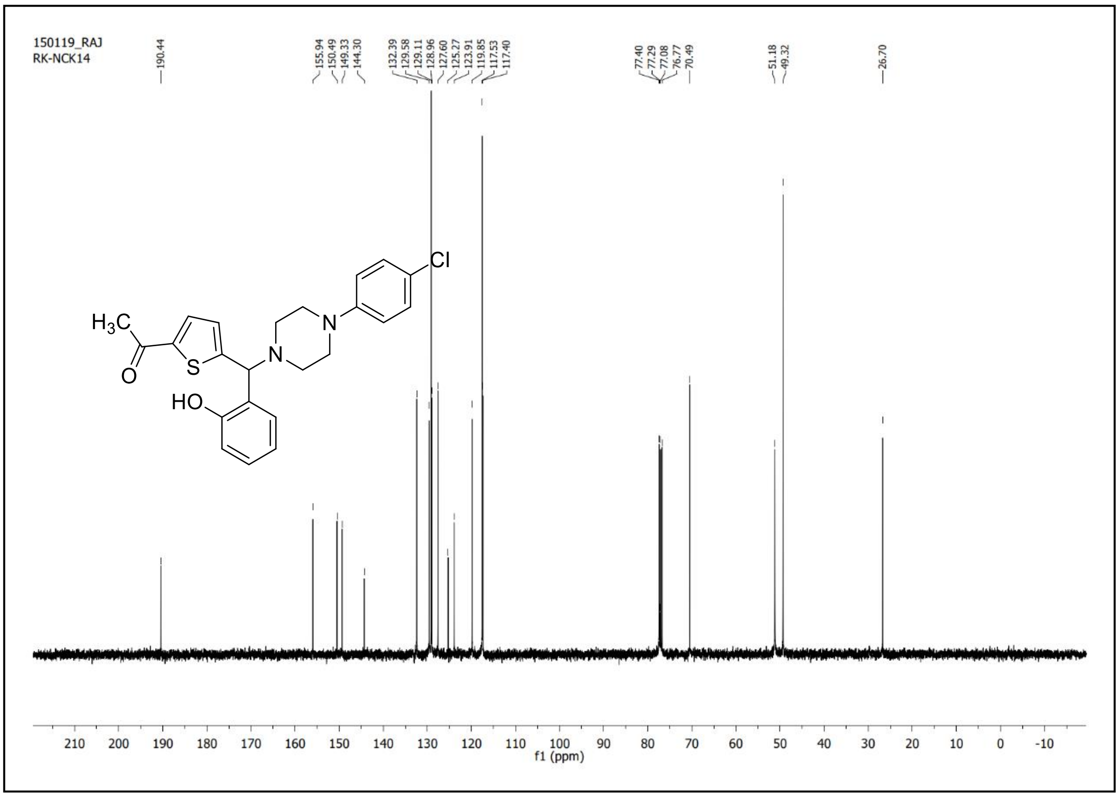


S41:$^{13}$C NMR spectrum of compound **4r**

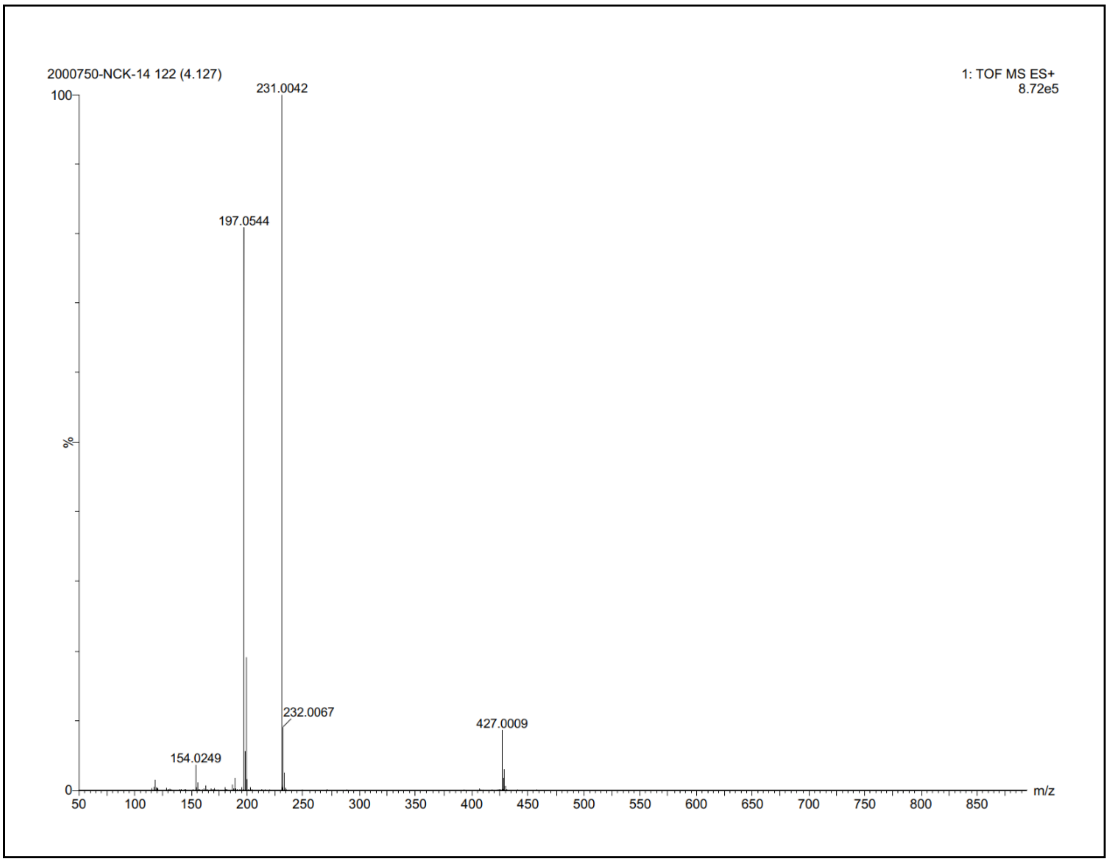


S42:Mass spectrum of compound **4r**

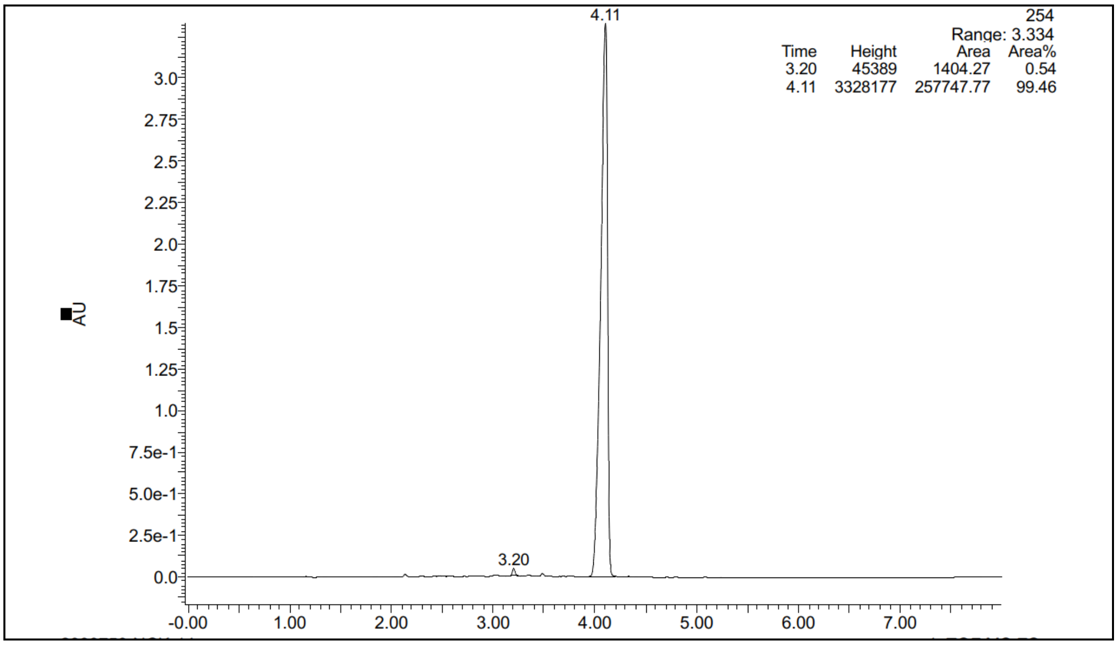


S43:LC Chromatogram of compound **4r**

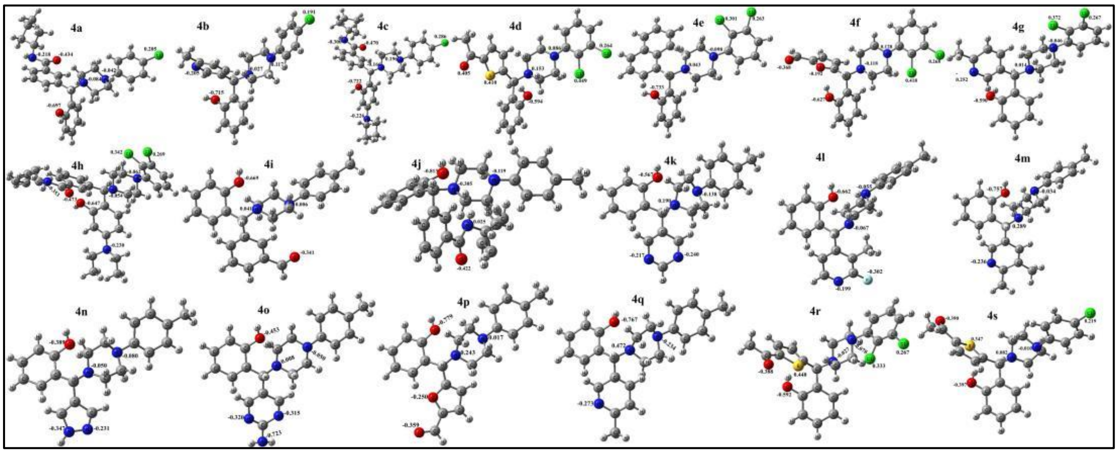

Figure S2. Optimized geometries of all the synthesized molecules studied in this work and charges (in e) of hetero atoms are given in the Figure.
